# LC‐IRMS Persulfate Oxidation: Case Study on Neonicotinoid‐Related Structures

**DOI:** 10.1002/rcm.10067

**Published:** 2025-05-12

**Authors:** Felix Niemann, Annika Gruhlke, Maik A. Jochmann, Torsten C. Schmidt

**Affiliations:** ^1^ Faculty of Chemistry, Instrumental Analytical Chemistry University of Duisburg‐Essen Essen Germany; ^2^ Centre for Water and Environmental Research (ZWU) Essen Germany; ^3^ IWW Water Centre Mülheim an der Ruhr Germany

**Keywords:** LC‐IRMS, neonicotinoids, oxidation Interface, persulfate

## Abstract

**Rationale:**

Liquid chromatography‐isotope ratio mass spectrometry (LC‐IRMS) is used to analyze stable carbon isotope ratios of polar nonvolatile compounds. However, challenges with the persulfate‐based oxidation interface have been reported, particularly for molecules with recalcitrant structures like those found in neonicotinoids. This study systematically investigates the oxidation efficiency of neonicotinoid‐related structures in a commercial LC‐IRMS.

**Methods:**

Neonicotinoid proxies of varying molecular complexity were evaluated for carbon recovery and stable carbon isotope ratio accuracy. LC‐IRMS parameters such as oxidant concentration, reaction time, temperature, acid concentration, and the presence of AgNO_3_ catalyst were varied. Carbon recoveries and *δ* ^13^C biases were determined by injecting an oxidation‐independent inorganic carbon standard under identical conditions. Elemental analyzer isotope ratio mass spectrometry (EA‐IRMS) was used to normalize *δ* ^13^C values.

**Results:**

Several neonicotinoid derivatives exhibited low carbon recovery and significant *δ* ^13^C bias. Increasing oxidant concentration, reactor temperature, and reaction time improved recoveries but did not fully mitigate isotopic biases. The addition of AgNO_3_ improved carbon recoveries for most derivatives but introduced variability in *δ* ^13^C values, likely due to shifts in reaction mechanisms. A workflow to identify oxidation problems during method development was proposed.

**Conclusions:**

Optimization of LC‐IRMS oxidation parameters is critical for urea, guanidine, and nitroguanidine derivatives and similar compounds. A systematic evaluation of oxidation efficiencies under different conditions is needed for optimal mineralization and thus more accurate *δ* ^13^C ratios.

## 1 Introduction

Compound‐specific stable isotope analysis (CSIA) quantifies the stable isotope ratios of individual elements in specific organic compounds in complex matrices. Currently, there is a lack of CSIA instrumentation and methods for nonvolatile substances. For volatile substances or those with suitable derivatization protocols, gas chromatography‐isotope ratio mass spectrometry (GC‐IRMS) is the preferred option for various elements (H, C, N, O, and Cl) [1, 2]. The commercialization of liquid chromatography‐isotope ratio mass spectrometry (LC‐IRMS) in 2004 was intended to address the aforementioned gap for stable carbon isotopes [3]. The method is based on an aqueous chromatographic separation and consecutive mixing with *ortho*‐phosphoric acid (H_3_PO_4_) and salts of S_2_O_8_^2−^ (peroxydisulfate, persulfate, and PDS) in a mixing tee. The combined stream is directed through a metal capillary that is coiled around a heating element, which facilitates the chemical oxidation of each compound to CO_2_. The resulting CO_2_ is transferred from the aqueous phase by a gas‐permeable membrane into a helium stream and is finally analyzed by an IRMS. Since its commercialization, the system has been successfully employed in the analysis of a range of analytes, including sugars, organic acids, amino acids, pharmaceuticals, and pesticides [4, 5, 6, 7].

It should be noted that the use of LC‐IRMS is not without certain confinements and drawbacks. Firstly, the use of organic eluents is restricted by the oxidation of any organic matter present in the combustion interface. Therefore, the separation in a fully aqueous eluent is controlled by temperature, inorganic modifiers, and pH gradients [8]. Because of their better compatibility with water, stationary phases with ion exchange capabilities are preferred over reversed‐phase columns [9, 10]. Secondly, the chromatographic resolution is significantly diminished by the lengthy run times, substantial dead volume of the system, and the transfer of CO_2_ into the gas phase. In addition, it is important to consider the potential impact of unsuitable PDS oxidation conditions as a source of error in carbon isotope ratio analysis [11].

The oxidation of organic molecules by PDS can occur via radical and nonradical pathways and is highly dependent on pH, temperature, present ions, and their concentrations. Despite its high reduction potential *E* ^0^_acidic_(S_2_O_8_^2−^/HSO_4_^−^) = +2.12 V [12], the S_2_O_8_^2−^ anion itself shows rather low reactivity with most compounds [13, 14]. Consequently, some form of activation is usually required. In the LC‐IRMS interface, the reaction conditions are characterized by high PDS concentrations (10–220 mM), acidic pH < 2, high concentrations of H_3_PO_4_ and H_2_PO_4_^−^ (p*K*_a_ = 2.15), and high temperatures (≈100°C) [15]. The following reactions should provide a qualitative understanding of the main processes occurring in the LC‐IRMS oxidation interface. Sulfate radicals (SO_4_•^−^) are the dominant radical species under acidic pH conditions *E* ^0^(SO_4_•^−^/SO_4_^2−^) = +2.44 V [16, 17]. It is assumed that PDS is activated via a solely temperature‐dependent homolytic bond cleavage (Equation (1)) and an acid‐catalyzed pathway (Equation (2)). The latter leads to the formation of persulfuric acid (Equations (3) and (4)) (H_2_SO_5_, also known as Caro's acid) *E* ^0^_acdic_(HSO_5_^−^/HSO_4_^−^) = +1.81 V [18].

(1) $${S_{2}O_{8}}^{2-}\to 2{\mathrm{SO}}_{4}\cdot^{-}$$

(2) $${S_{2}O_{8}}^{2-}+H^{+}\to {{\mathrm{HS}}_{2}O_{8}}^{-}$$

(3) $${{\mathrm{HS}}_{2}O_{8}}^{-}+H_{2}O\overset{\text{fast}}{\to }H{{\mathrm{SO}}_{4}}^{-}+H_{2}{\mathrm{SO}}_{5}$$

(4) $$H_{2}{\mathrm{SO}}_{5}\overset{\;\text{fast}}{\to }H^{+}+{{\mathrm{HSO}}_{5}}^{-}$$

The reaction of H_2_PO_4_^−^ and SO_4_•^−^ is slow and might only take place to a small extent (Equation (5)) [19]. Due to the high PDS concentrations, sulfate radical recombination (Equation (6)) and self‐quenching (Equation (7)) might occur [20].

(5) $${H_{2}{\mathrm{PO}}_{4}}^{-}+{\mathrm{SO}}_{4}\cdot^{-}\to H_{2}{\mathrm{PO}}_{4}\cdot +{{\mathrm{SO}}_{4}}^{2-}$$

(6) $${\mathrm{SO}}_{4}\cdot^{-}+{\mathrm{SO}}_{4}\cdot^{-}\to {S_{2}O_{8}}^{2-}$$

(7) $${\mathrm{SO}}_{4}\cdot^{-}+{S_{2}O_{8}}^{2-}\to {{\mathrm{SO}}_{4}}^{2-}+S_{2}O_{8}\cdot^{-}$$

One challenge inherent to LC‐IRMS is posed by the “unproductive” PDS hydrolysis, which results in the formation of O_2_. This phenomenon is pH dependent and proceeds over multiple intermediates but can be described by the stoichiometry of Equation (8) [21].

(8) $${S_{2}O_{8}}^{2-}+H_{2}O\to 2{{\mathrm{SO}}_{4}}^{2-}+\frac{1}{2}O_{2}+2H^{+}$$

The addition of Ag^+^ salts to the H_3_PO_4_ is sometimes practiced to facilitate the formation of sulfate radicals (Equation (9)) but can also lead to SO_4_•^−^ quenching (Equation (10)). Ag^2+^ itself is an oxidant with a different selectivity than SO_4_•^−^ *E* ^0^(Ag^2+^/Ag^+^) = +1.98 V [22, 23]. Subsequent reduction of Ag^2+^ back to Ag^+^, for example, by organics, closes the catalytic cycle. Preliminary tests with other transition metal catalysts did not indicate potential for LC‐IRMS application [24].

(9) $${S_{2}O_{8}}^{2-}+{\mathrm{Ag}}^{+}\to {{\mathrm{SO}}_{4}}^{2-}+{\mathrm{SO}}_{4}\cdot^{-}+{\mathrm{Ag}}^{2+}$$

(10) $${\mathrm{SO}}_{4}\cdot^{-}+{\mathrm{Ag}}^{+}\to {{\mathrm{SO}}_{4}}^{2-}+{\mathrm{Ag}}^{2+}$$

In the context of LC‐IRMS and its instrumentation, several studies have been conducted on the oxidation by PDS. Gilevska et al. conducted an investigation of halogenated acetic acids and substituted aromatic compounds, revealing that the lowest recoveries were observed for multiple fluorinated and chlorinated acetic acids [24]. Incomplete oxidation was also reported for caffeine, yet a method to distinguish between natural and synthetic sources was developed [25, 26]. Similar challenges were described for bentazone [27], sulfonamides [8], and natural organic matter [28]. Diaz et al. observed incomplete oxidation by PDS of substances with conjugated C=N bonds, with those containing guanidinium‐like structures exhibiting particularly low carbon recoveries [29].

**FIGURE 1:**
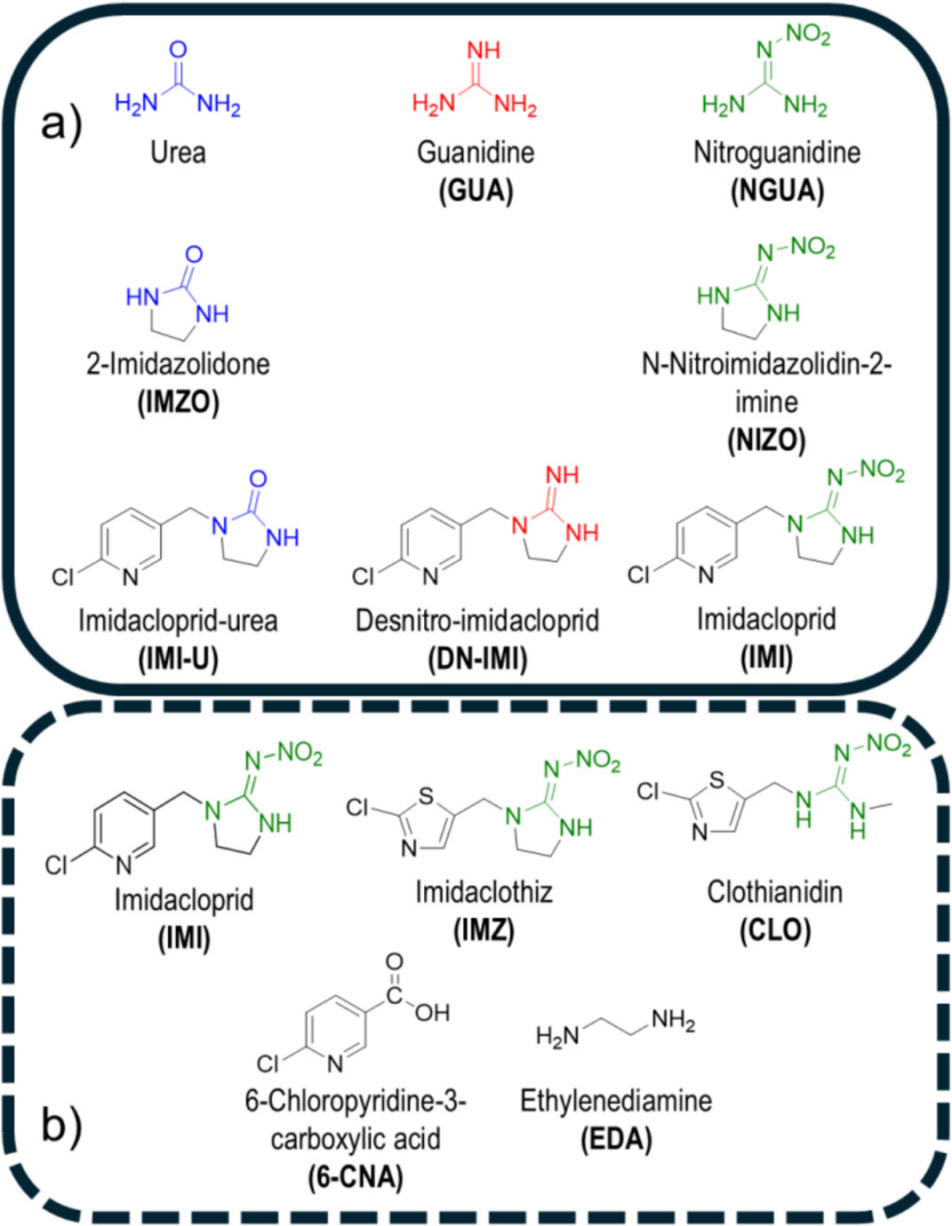
The nitroguanidine neonicotinoid‐related structures selected for the LC‐IRMS oxidation interface performance study. Panel (a) illustrates urea, guanidine and nitroguanidine derivatives with increasing structural complexity. Panel (b) shows the investigated nitroguanidine neonicotinoids and 6‐chloropyridine‐3‐carboxylic acid serving as a proxy for the chloropyridine group. Ethylenediamine has been studied as part of the imidazolidine ring.

These structures are also present in nitroguanidine neonicotinoid insecticides, including clothianidin, imidacloprid, and imidaclothiz, as well as their environmental degradation products. We recently published a study on imidacloprid degradation by hydrolysis and photolysis using LC‐IRMS which prompted the idea for an in‐depth look into the performance of the wet persulfate‐based oxidation interface [30]. As those developing LC‐IRMS methods that are required to evaluate the oxidation performance of compounds of interest, this study aims to systematically investigate the influence of all system parameters affecting wet‐PDS‐based oxidation on carbon recoveries and *δ* ^13^C value accuracies in a systematic manner. We selected proxies with simple structures, including urea, guanidine, and nitroguanidine, as well as neonicotinoid‐derived structures containing them in a molecular framework (see Figure 1). Based on the insights gained from this case study on neonicotinoid‐related structures and existing literature, we propose general recommendations for LC‐IRMS method developers.

## 2 Experimental Section

### 2.1 Chemicals and Reagents

A comprehensive overview of all chemicals, suppliers, and considerations regarding standard preparation and storage can be found in section S1 of the Supporting Information. All standards and reagents were prepared in ultrapure water (> 18 MΩ) provided by an Arium Pro VF (Sartorius Lab Instruments, Göttingen, Germany).

### 2.2 Isotope Analysis

All proxy compounds and laboratory standards were measured by an elemental analyzer coupled to an isotope ratio mass spectrometer (EA‐IRMS). The system used was a Pyrocube coupled to an Isoprime 100 (both Elementar Analysensysteme, Langenselbold, Germany). An internal acetanilide standard was measured repetitively to monitor *δ* ^13^C stability. Normalization to the VPDB scale was achieved through 2‐point calibration of the international reference materials USGS40 (*δ* ^13^C_VPDB_ = −26.39 ± 0.04‰) [31] and 41a (*δ* ^13^C_VPDB_ = 36.55 ± 0.08‰) [32] (both Reston, USA). Further details on EA‐IRMS measurements can be found in section S2 of the Supporting Information.

**FIGURE 2:**
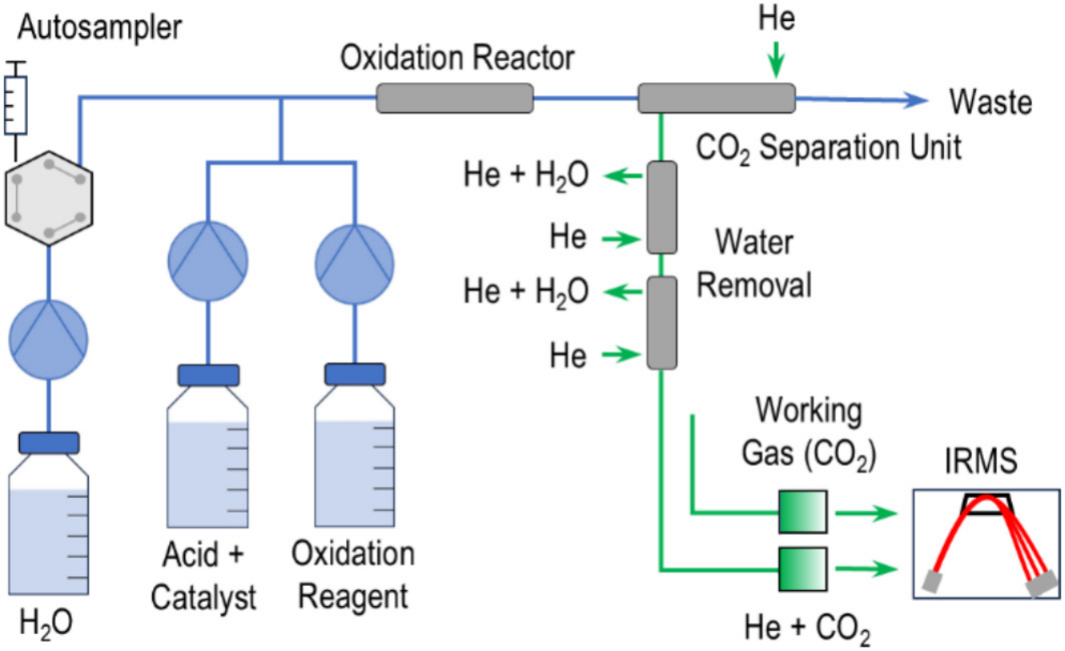
LC‐IRMS instrumentation in flow injection (μ‐EA mode). Blue is indicative of the liquid phase, while green is indicative of the gas phase.

LC‐IRMS measurements were performed on an LC‐Isolink coupled to a DeltaV Advantage (both Thermo Fisher Scientific, Bremen, Germany) in flow injection mode (μEA‐mode) (see Figure 2). A Dionex Ultimate 3000 (Thermo Fisher Scientific, Sunnyvale, USA) was used for H_2_O eluent delivery and sample injection. Two reagent pumps delivered H_3_PO_4_ and sodium persulfate oxidizing agent (Na_2_S_2_O_8_). In experiments with silver nitrate (AgNO_3_) metal catalyst, it was added to the H_3_PO_4_. Eluents and reagents were degassed for 15 min under vacuum in a Sonorex Digitex ultrasonic bath (Bandelin, Berlin, Germany) to remove dissolved atmospheric CO_2_. Furthermore, reagents were kept in amber glass bottles to improve stability. The reagent and eluent streams were combined by a T‐piece and transferred to a temperature‐controlled heated steel capillary for analyte oxidation to CO_2_. The resulting gas was then transferred into a helium stream through a gas‐permeable membrane. The gas stream was dried sequentially through two Nafion membranes prior to its introduction into the IRMS via an open split. An additional open split was employed for the programmable introduction of CO_2_ working gas pulses.

### 2.3 LC‐IRMS Oxidation Efficiency Assessment

The performance of the LC‐IRMS oxidation interface was evaluated for 14 neonicotinoid‐related proxy compounds. Each instrumental parameter affecting oxidation, namely, reaction time/total flow rate, oxidant concentration, temperature, acid concentration, or metal catalyst addition was varied stepwise and individually from selected reference conditions (Ref). This approach enabled the evaluation of the influence of each parameter on each compound to be assessed individually. The reference conditions were HPLC, acid, and oxidant flows of 500, 50, and 50 μL min^−1^, respectively. The heated capillary was kept at a standard value of 100°C [33]. H_3_PO_4_ and oxidant concentrations were 1.5 M and 100 g L^−1^. This corresponds, after the dilution of oxidant by eluent and acid, to a final concentration of 35 mM of PDS in the reactor. According to an analysis of PDS concentrations used in 52 published LC‐IRMS methods, this value would fall into the lowest quartile [15].

Three criteria were selected as indicators of oxidation interface performance. The first criterion is the IRMS signal response of a compound in comparison to an internal sodium bicarbonate (NaHCO_3_) standard (Equation (10)) injected under identical instrumental conditions and with an identical injected carbon amount (18 nmol C corresponding to 5 μL injections of 43 mg L^−1^ C standards). It is assumed that NaHCO_3_ is quantitatively converted to CO_2_ under the acidic conditions present in the interface and thus serves as a reference point for 100% CO_2_ signal recovery. Further details on the selection and validation process of NaHCO_3_ as a reference substance can be found in the Supporting Information S3. The recorded peak area *A* is determined as the sum of the tree mass traces (*m/z* = 44, 45, and 46) with their respective amplification factors considered. The recovery *R* is determined using the ratio given by Equation (11).

(11) $$\frac{A_{\text{Compound}}}{A_{{\text{NaHCO}}_{3}}}=R_{\text{Compound}}$$

The second criterion evaluates the *δ* ^13^C bias introduced by the LC‐IRMS oxidation interface. Ideally, after normalization to the VPDB scale, the carbon isotope ratios measured by LC‐IRMS should align with those measured and normalized by EA‐IRMS. To achieve VPDB normalization for LC‐IRMS, two oxidation‐insensitive laboratory standards (oxalic acid and NaHCO₃) were first normalized to the VPDB scale using EA‐IRMS via a 2‐point calibration. Oxalic acid dihydrate (*δ* ^13^C_Oxalix Acid_ = −33.8 ± 1.7‰) and NaHCO_3_ (*δ* ^13^C_NaHCO3_ = −7.44 ± 0.1‰) were found to have sufficiently distinct carbon isotope ratios to account for scale nonlinearities inherent to the system.

These laboratory standards were then analyzed alongside the target compounds under each tested oxidation condition on the LC‐IRMS to calculate 2‐point normalized values (*δ* ^13^C_LC‐IRMS,Compound_). Deviations from the expected “true” value (*∆δ* ^13^C) determined by the EA‐IRMS (*δ* ^13^C_EA‐IRMS,Compound_) could be calculated according to Equation (12):

(12) $$∆\delta^{13}C=\delta^{13}C_{\mathrm{LC}-\text{IRMS},\text{Compound}}-\delta^{13}C_{\mathrm{EA}-\text{IRMS},\text{Compound}}$$

The third criterion for LC‐IRMS system performance is pointed towards concentration dependent effects. Carbon amounts from 9 to 72 nmol were injected to test the stability of *δ* ^13^C values in addition to linearity and slope of the signal area response. All measurements were done in triplicate injections, and respective errors are reported as ± 1 × σ.

## 3 Results and Discussion

### 3.1 LC‐IRMS Oxidation Interface Performance

The evaluation of oxidation efficiencies in the LC‐IRMS interface of selected neonicotinoid‐related compounds revealed significant challenges with incomplete carbon recoveries and *δ* ^13^C value bias. All instrumental parameters affecting the oxidation need careful optimization. Modifying these parameters has further implications for the LC‐IRMS system and methodology. The reaction time is dependent on the total flow, which is a combination of eluent, oxidant, and acid flows. If the instrument is not operated in μ‐EA mode, the flow requirements of the chromatographic separation must be met, which often limits the ability to reduce flows to increase reaction times. In this study, the total flow rates (*Q*) were maintained at constant ratios between the pumps. The reference flow rate *Q*_Ref_ was set to 600 μL min^−1^, and lower flow rates were expressed as percentages of *Q*_Ref_ (e.g., 40% *Q*_Ref_ = 240 μL min^−1^). Based on the volume of 196 mm^3^ for the reactor in the commercial system, determined by Köster et al., the corresponding reaction times were found to be 20 and 53 s, respectively [15]. The high salt concentrations and back pressure generated by the gas separator elevate the boiling point of water sufficiently to permit higher reaction temperatures. Elevated temperatures also result in increased unproductive persulfate hydrolysis and O₂ formation (Equation (8)). The measured O_2_ backgrounds *m/z* = 32 (3 × 10^8^ Ω) for 100°C, 105°C, and 110°C were 13, 22, and 36 V, respectively. A change in PDS concentration from 100 to 200 g L^−1^ resulted in an increase in O_2_ backgrounds from 13 to 24 V. An overview of background signals can be found in Table S3 in the Supporting Information. The removal of excess O_2_ can be achieved in the gas phase through the use of a regeneratable copper reactor, as previously described by Hettmann et al. [34].

Clogging of in‐line filters or the gas separator can be a major issue especially when using AgNO_3_. Large amounts of sulfide or halogens, especially Cl^−^, Br^−^, and I^−^ must be kept out of the flow line, as they form insoluble silver salts. When the interface pumps were operated at low flow rates, particularly in standby mode, the presence of a yellow precipitate of a silver salt was observed at the purge valve. Similar issues have been reported in the past [24]. Therefore, regular flushing with water, especially in standby mode, is essential for AgNO_3_ users to avoid clogging of in‐line filters and the gas separation membrane. The pH affects the oxidation and potentially the transfer of formed CO_2_ across the gas separation membrane. The pH of the effluent is not the same as the pH at which the reaction occurs due to temperature‐dependent dissociation of H_3_PO_4_. Furthermore, unproductive PDS decomposition (Equation (8)) results in acidification even without acid addition. The pH values of the effluent were measured for all experimental conditions and are presented in Table S2 of the Supporting Information. The measured values ranged from 1.3 for the use of 3 M H_3_PO_4_, 1.5 for the reference conditions, and 2.4 when H_2_O was pumped instead of acid. All measured pH values should be sufficiently low to facilitate the quantitative conversion of dissolved inorganic carbon to CO_2_ (p*K*_a_ = 6.35) [12], which is a prerequisite for transport across the gas‐permeable membrane. The absence of H_3_PO_4_ could potentially increase the susceptibility of the system to corrosion over time, as it acts as a passivating agent for the stainless‐steel capillaries. However, no corrosion was observed during our experiments.

**FIGURE 3:**
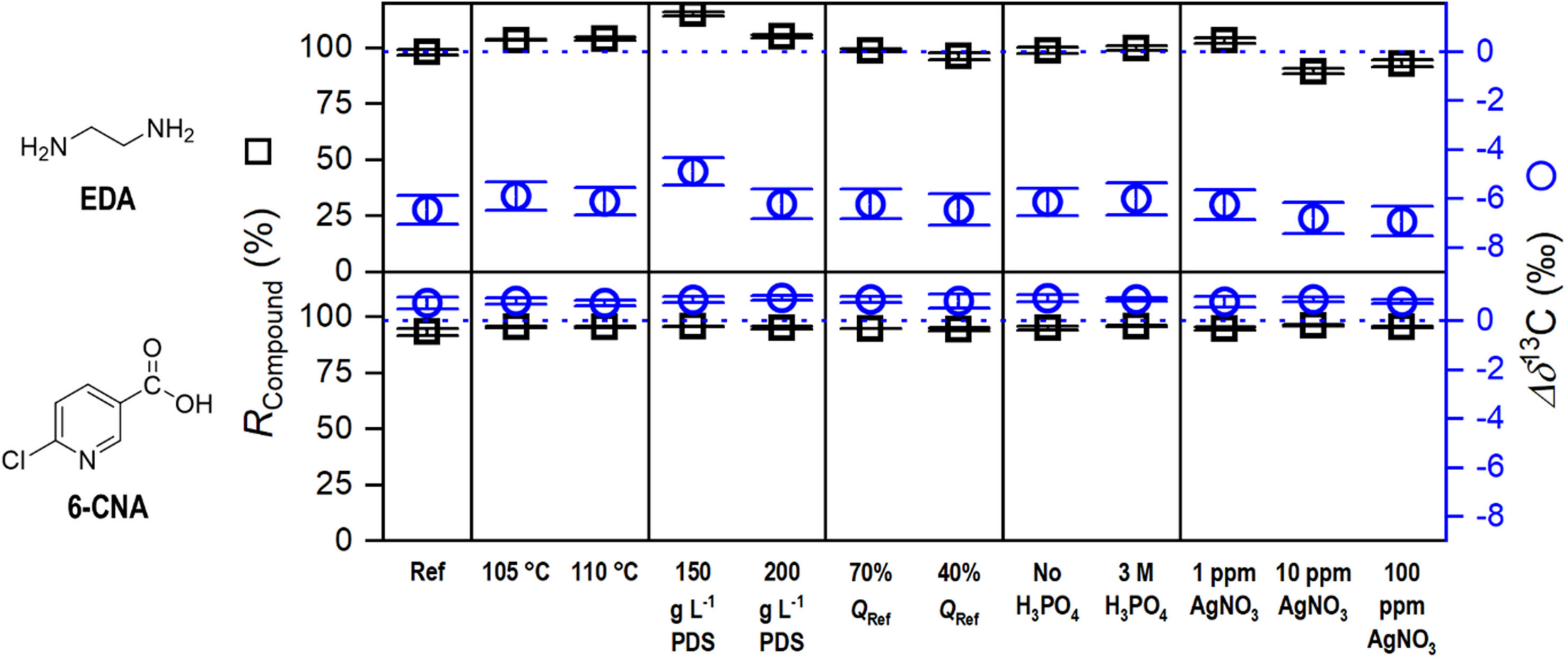
LC‐IRMS oxidation unit performance for ethylenediamine and 6‐chloronicotinic acid. Reference conditions (Ref) use flowrates of 500, 50, and 50 μL min^−1^ for eluent, oxidation agent, and acid pumps, respectively (*Q*_Ref_ = 600 μL min^−1^), 100°C reactor temperature, an oxidant concentration of 100 g L^−1^ Na_2_S_2_O_8_, an H_3_PO_4_ acid reagent concentration of 1.5 M and no AgNO_3_. *X*‐axis labeling refers to a varied parameter with respect to the reference conditions. Blue dotted line serves as a *∆δ* ^13^C = 0‰ reference line equaling to no deviation from EA‐IRMS values.

**FIGURE 4:**
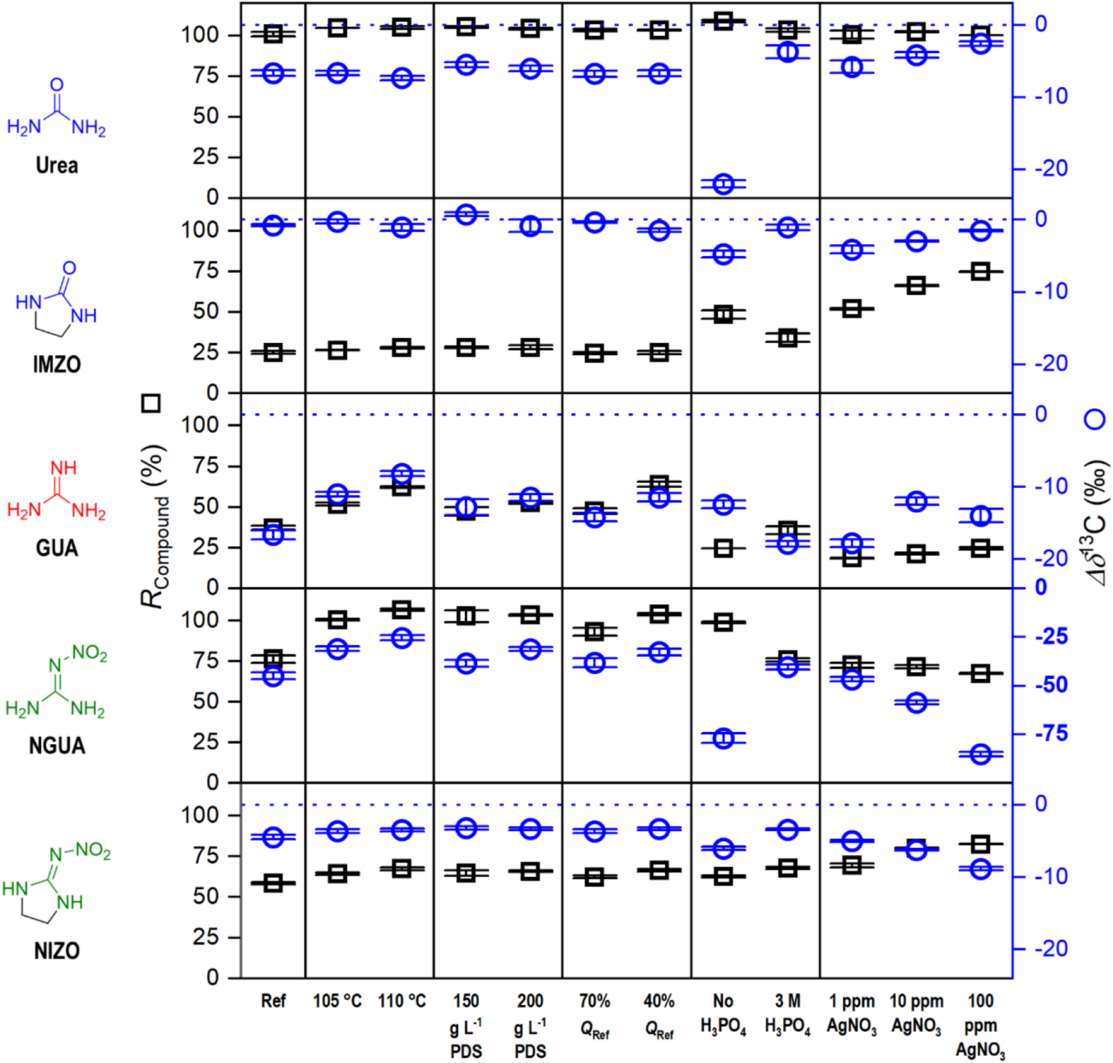
LC‐IRMS oxidation unit performance for small critical structures related to neonicotinoid pesticides. Reference conditions (Ref) use flowrates of 500, 50, and 50 μL min^−1^ for eluent, oxidation agent, and acid pumps, respectively (*Q*_Ref_ = 600 μL min^−1^), 100°C reactor temperature, an oxidant concentration of 100 g L^−1^ Na_2_S_2_O_8_, an H_3_PO_4_ acid reagent concentration of 1.5 M, and no AgNO_3_. *X*‐axis labeling refers to a varied parameter with respect to the reference conditions. Blue dotted line serves as a *∆δ* ^13^C = 0‰ reference line equaling to no deviation from EA‐IRMS values.

**FIGURE 5:**
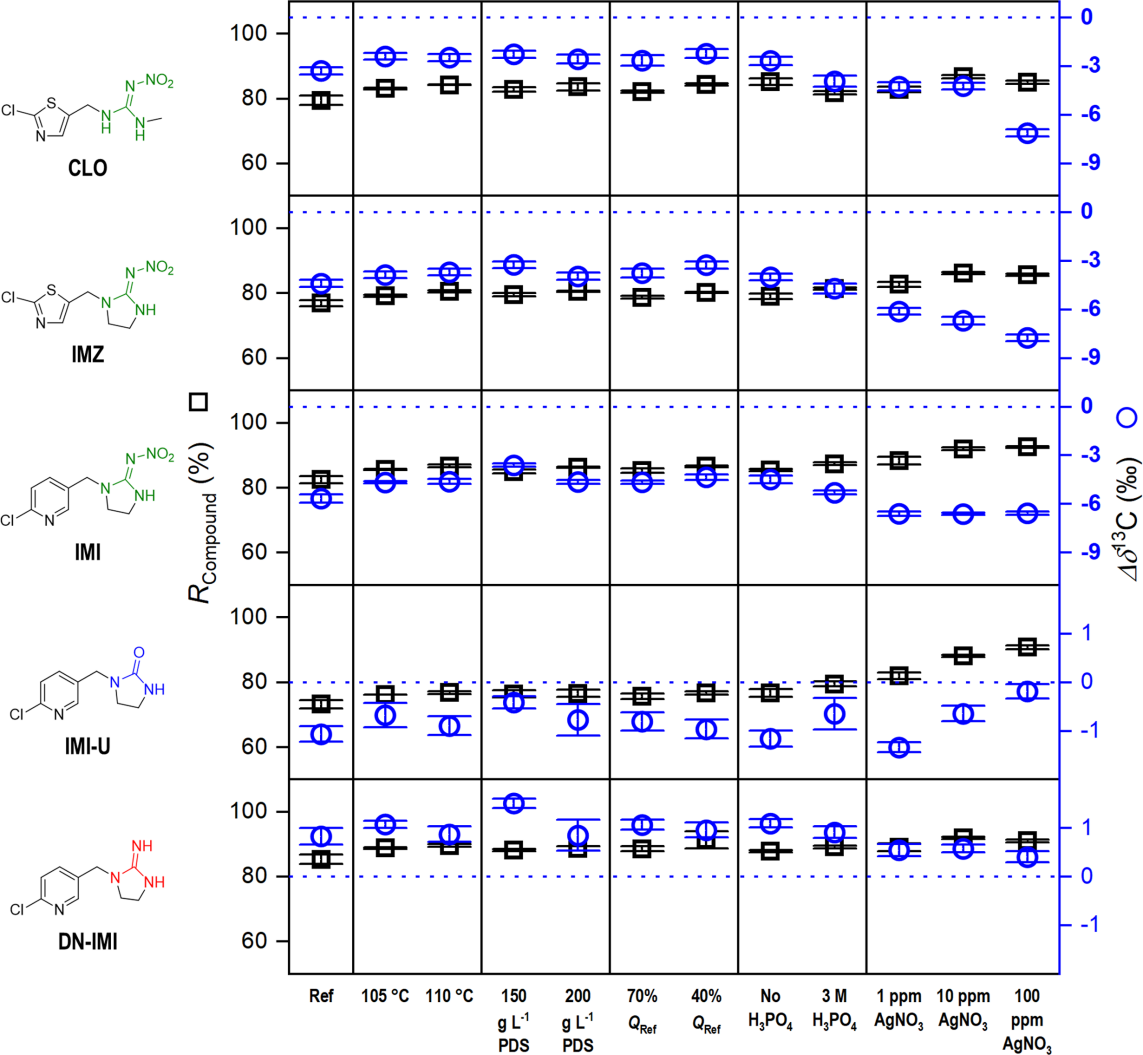
LC‐IRMS oxidation unit performance for the nitroguanidine neonicotinoids imidacloprid, imidaclothiz, and clothianidin and the imidacloprid transformation products imidacloprid‐urea and desnitro‐imidacloprid. Reference conditions (Ref) use flowrates of 500, 50, and 50 μL min^−1^ for eluent, oxidation agent, and acid pumps, respectively (*Q*_Ref_ = 600 μL min^−1^), 100°C reactor temperature, an oxidant concentration of 100 g L^−1^ Na_2_S_2_O_8_, an H_3_PO_4_ acid reagent concentration of 1.5 M and no catalyst. *X*‐axis labeling refers to a varied parameter with respect to the reference conditions. Blue dotted line serves as a *∆δ* ^13^C = 0‰ reference line equaling to no deviation from EA‐IRMS values.

The first two LC‐IRMS oxidation performance criteria, carbon recovery and *∆δ* ^13^C relative to an NaHCO_3_ standard injected under identical conditions, were evaluated. Figures 3 and 4 show the results for all oxidation‐critical neonicotinoid subunits, while Figure 5 shows the results for the entire neonicotinoids and the two common IMI degradation products, DN‐IMI and IMI‐U. In cases where a high conversion rate to CO_2_ has been observed, a low *∆δ* ^13^C would be expected, but this has not been consistently seen. The results demonstrate a notable deviation from the anticipated value of 0‰ for EDA, GUA, and all NGUA derivatives, including the neonicotinoids. Given that the offset sometimes occurs despite high C‐recoveries, we hypothesize that the isotopic bias may be attributed to the contribution of formed N_2_O or NO_2_. The idea of possible bias by introduction of nitrogenous species has been mentioned previously when using (NH_4_)_2_S_2_O_8_ instead of Na_2_S_2_O_8_ as an oxidation reagent [35]. PDS oxidation of nitrogenous organic compounds forms mineralization products such as NH_4_^+^ and NO_3_^−^ as a function of pH [36]. HNO_2_, a possible intermediate, might disproportionate to NO and NO_2_ (*m/z* = 46) in acidic environment [37]. Such an effect holds theoretically potential to bias *δ* ^13^C values, but its significance in LC‐IRMS has not been systematically investigated yet. Installing the previously mentioned copper reactors could avoid such complications by converting N_x_O_y_ to N_2_.

To identify structural features of recalcitrant neonicotinoid derivatives, we examined the chloropyridine and imidazolidine moieties isolated. The 6‐CNA results show high recoveries under all tested conditions. A systematic study employing a comparable reactor revealed that nicotinic acid, the unchlorinated derivative of 6‐CNA, exhibited notable recalcitrance in acidic and alkaline PDS oxidation [38]. The ‐I‐effect of substituted chlorine in 6‐CNA should result in the deactivation of the aromatic ring for SO_4_•^−^ attack. On the other hand, it increases the acidity of the pyridinium cation. This potentially facilitated oxidation as the reaction of pyridinium with sulfate radicals is known to be slower than that of unprotonated pyridine [39]. Similarly, in 6‐chloronicotine‐neonicotinoid derivatives, protonation does not occur for the same reason, indicating that the 6‐chloropyridine group of IMI, IMI‐U, and DN‐IMI is not recalcitrant either.

Therefore, urea, GUA, and NGUA were investigated individually, and urea and NGUA were also examined in their ethylene‐bridged form. Complete carbon recovery was only achieved for urea, regardless of the oxidation conditions employed, and for NGUA with increased reaction temperature, residence time, oxidant concentration, or without H_3_PO_4_. The ethylene bridge reduced carbon recoveries except if AgNO_3_ was added. Interestingly, the addition of AgNO_3_ had opposite effects for GUA and NGUA with and without ethylene bridging. A recovery enhancement resulting from the addition of AgNO_3_ was also observed in the case of IMI‐U which is consistent with the improvement observed for IMZO by the addition of AgNO_3_, suggesting that this imidazolidine derived moiety is indeed the recalcitrant part of the neonicotinoids and their transformation products. A similar link can be made between the result of AgNO_3_ addition to NIZO and the nitroguanidine neonicotinoids containing this structure.

The nitroguanidine neonicotinoids CLO, IMZ, IMI, and the IMI transformation products IMI‐U and DN‐IMI exhibited recoveries between 75% and 92%. An increase in temperature, PDS concentration, and residence time resulted in enhanced recovery. Increasing these instrumental parameters lowered the *∆δ* ^13^C, although not substantial. Apart from DN‐IMI and IMI‐U, all substances exhibited a *∆δ* ^13^C ≥ 1‰ up to 8‰. The addition of AgNO_3_ for nitroguanidine neonicotinoids follows a similar trend to that observed for the proxy structures NGUA and NIZO, whereby although carbon recoveries are rising, the *∆δ* ^13^C value is decreasing. In conclusion, it can be stated that LC‐IRMS methods for neonicotinoid derivatives need careful selection of oxidation parameters. The simultaneous alteration of parameters such as oxidant and AgNO_3_ concentration was not carried out in the present study but should lead to near quantitative (> 90%) conversion rates for the investigated neonicotinoid derivatives.

Increasing the reaction temperature, oxidant concentration, or reaction time (decreasing the flow rate) led to expected positive effects on carbon recoveries and *∆δ* ^13^C values. The extent of this effect was found to depend on the specific substance in question. It could increase, sometimes double, the recoveries of GUA and NGUA, whereas the effect on IMZO and NIZO was not pronounced. *∆δ* ^13^C values were either unaffected or improved towards 0‰. This illustrates that increasing oxidant concentration, temperature, and reaction time alone does not always result in sufficient carbon mineralization for isotope analysis. However, changing the concentration of H_3_PO_4_ or introducing AgNO_3_ can fundamentally change the reactivity. In the absence of H_3_PO_4_, only the recoveries of GUA showed a slight decrease. The recoveries of all other substances remained constant or increased in the case of NGUA. The absence of H_3_PO_4_ increased the *∆δ* ^13^C values of urea, IMZO, NGUA, and NIZO. This effect did not correlate with a decreased C recovery, so it remains unclear whether this effect is related to oxidation or a decreased efficiency of CO_2_ membrane transport. Despite the measured effluent pH of 2.4, there may be locally higher values around the analyte peak. The addition of 3 M H_3_PO_4_ did not significantly increase the recovery of most substances, except for a slight increase observed for NIZO and IMZO. This suggests that there are other effects than just increased persulfate activation by the acid‐catalyzed pathway at play, and pH needs to be optimized empirically for each substance. The quenching of SO_4_•^−^ by H_2_PO_4_^−^ according to Equation (5) with the resulting formation of H_2_PO_4_• radicals may be responsible for the decreased mineralization. We suspect the formation of reactive species with different selectivity after AgNO_3_ addition such as Ag^2+^ (Equation (9)). Also, AgNO_3_ concentration should be kept constant once a method is developed. Activation and quenching reactions such as Equations (9) and (10) dictate an optimal concentration range, which we found in preliminary experiments to be ≤ 400 mg L^−1^ AgNO_3_ for our instrumental conditions. The introduction of AgNO_3_ led to an improvement in the recovery of all substances, except for GUA and NGUA, where it resulted in a decline. In addition to the mere presence of AgNO_3_, its concentration also influenced recoveries and *δ* ^13^C values.

The third performance criterion, the linearity of detector response and concentration independence of *δ* ^13^C‐values, was determined by injecting carbon amounts from 9 to 72 nmol. The complete results can be found in Section S5 of the Supporting Information. The slopes obtained by linear regression for the detector response are summarized in Table 1. We confirm the finding that a linear detector response and a good coefficient of determination (*R* ^2^) of a compound alone does not always correspond to high conversion rates [15]. The lowest *R* ^2^ value of 0.895 was observed for GUA with 100 ppm added AgNO_3_ but many analytes where the other criteria clearly indicate insufficient oxidation show *R* ^2^ values > 0.999. And an interesting observation, not always well described by coefficients of determination, is a drop of residuals at higher concentration and the formation of a “plateau”. This does not occur with readily oxidizable substances, but the absence of a “plateau” is not a sufficient criterion to rule out recalcitrance. However, the slope itself is a good proxy for oxidation efficiency but needs some kind of reference of a “good” slope to be used as a criterion for sufficient oxidation. In μ‐EA mode, it can be directly compared to an IC standard, as there is no chromatographic effect involved. When developing an LC‐IRMS method, parameters at which *δ* ^13^C‐values are concentration independent should be found. Our data indicate that constant *δ* ^13^C values alone are also not sufficient to identify poor oxidation conditions, but if they are shifting with increasing concentrations, insufficient oxidation is a probable cause.

**TABLE 1:** Slopes of linear regressions for peak area detector response for each model compound under varying oxidation conditions.

	Normalized slope to NaHCO_3_					
Compound	Ref	40% *Q*_Ref_	110°C	200 g L^−1^ PDS	No H_3_PO_4_	100 ppm AgNO_3_
NaHCO_3_	100.0 ± 1.1	100.0 ± 3.2	100.0 ± 0.1	100.0 ± 0.2	100.0 ± 0.3	100.0 ± 0.5
IMZO	18.0 ± 0.3	21.6 ± 0.9	22.0 ± 0.2	19.2 ± 1.0	24.5 ± 2.7	73.0 ± 3.1
6‐CNA	93.4 ± 1.5	94.2 ± 3.3	95.2 ± 0.2	94.2 ± 0.1	93.8 ± 0.2	95.7 ± 0.2
CLO	79.9 ± 0.9	87.4 ± 2.5	84.0 ± 0.5	82.6 ± 0.2	82.6 ± 0.5	75.4 ± 5.0
DN‐IMI	86.4 ± 1.7	90.9 ± 2.1	92.0 ± 0.4	89.8 ± 0.4	85.5 ± 0.6	87.4 ± 3.2
EDA	112.5 ± 2.8	115.2 ± 4.6	108.5 ± 1.7	106.3 ± 0.4	101.8 ± 0.4	98.0 ± 1.2
GUA	21.0 ± 3.2	45.8 ± 7.4	58.7 ± 5.0	43.2 ± 8.0	21.2 ± 1.5	1.5 ± 0.5
IMI	82.7 ± 2.1	87.6 ± 2.4	87.9 ± 0.5	85.9 ± 0.3	82.9 ± 0.4	90.4 ± 1.9
IMI‐U	73.6 ± 0.8	76.2 ± 3.6	78.1 ± 0.5	77.0 ± 0.4	73.8 ± 0.3	89.2 ± 0.9
IMZ	77.1 ± 1.2	78.9 ± 4.2	82.3 ± 0.4	80.3 ± 0.1	76.6 ± 0.6	79.5 ± 4.7
NIZO	50.6 ± 2.2	61.4 ± 2.4	63.8 ± 1.1	60.1 ± 1.5	60.0 ± 0.3	73.6 ± 4.1
NGUA	35.6 ± 4.2	89.6 ± 8.4	107.0 ± 1.0	78.8 ± 14.5	63.6 ± 7.7	32.3 ± 8.8
Urea	98.6 ± 3.2	104.5 ± 2.8	105.3 ± 0.2	103.1 ± 0.2	103.0 ± 1.2	83.3 ± 8.8

**FIGURE 6:**
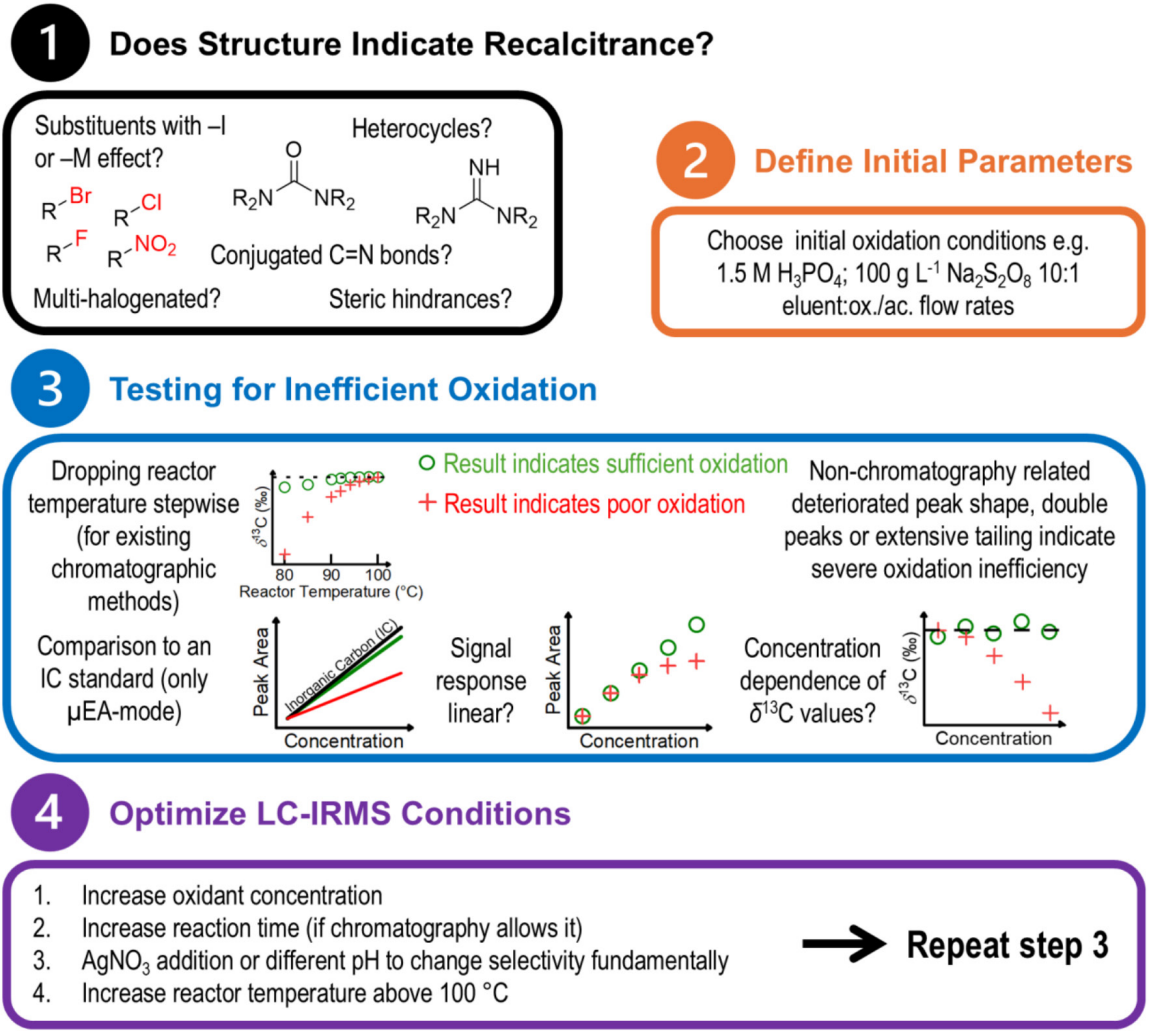
Suggested workflow to assess and optimize oxidation efficiency in LC‐IRMS methods.

The conversion of neonicotinoid‐related compounds to CO_2_ using the LC‐IRMS interface is challenging, and it illustrates the difficulties that method developers may face with other recalcitrant analytes. We have combined known procedures with the presented data to propose a workflow for method developers interested in a new compound (Figure 6). Firstly, certain structures in the analyte of interest may indicate recalcitrance such as conjugated C=N systems, guanidine moieties [29], multiple halogenation [24], steric hindrances, and substituents with ‐I or ‐M effects [40]. If such structures are not present, it is advisable to start with mild oxidation conditions to keep the amount of O_2_ in the ion source low and to prolong the filament lifetime [15, 33]. The manufacturer suggests that peak shape characteristics such as double peaks, peak broadening, and long tailing may indicate inadequate oxidation conditions [33]. We found this to be true only in very severe cases. Established tests for incomplete oxidation involve comparison of peak areas and *δ* ^13^C values of an analyte with an inorganic or readily oxidizable organic standard [15, 24, 29]. These can be conveniently carried out in μ‐EA mode and allow quick optimization of instrumental parameters. In an actual LC‐IRMS method using a chromatographic column, dilution of the analyte through peak broadening and possibly column material competing for oxidant may affect mineralization [25]. Gradually lowering the reactor temperature after analyte injection, while observing the peak area and *δ* ^13^C response, serves as a convenient tool in such situations, although there is no clear threshold for when a method does not provide sufficient oxidation conditions [15]. If long reaction times are required, this can be considered when selecting a column, as smaller ID columns have lower optimal flows and therefore longer residence times in the reactor can be achieved. If increasing the oxidant concentration alone is not sufficient, increasing the reactor temperature may be a suitable option, as it appears to provide the same improvements expected from increasing the PDS concentration but is not limited by solubility. Lower pH does not automatically equal more efficient mineralization. The relationship of pH and mineralization is rather complex and needs to be empirically optimized for recalcitrant compounds. The addition of AgNO_3_ catalyst should only be done if other options are not sufficient as it can increase the maintenance frequency while not always yielding improvements. It can however change the selectivity of the oxidation process and serve as an option for otherwise recalcitrant substances.

## 4 Conclusion

Sufficient, ideally quantitative conversion of analytes to CO_2_ in the wet persulfate‐based LC‐IRMS interface is a prerequisite for accurate *δ* ^13^C measurements. Recalcitrance was observed for urea, guanidine, and nitroguanidine derivatives, including nitroguanidine neonicotinoids and imidacloprid transformation products. The effect on *δ* ^13^C values and carbon recoveries was evaluated for each compound while varying instrumental parameters influencing the oxidation stepwise. Increasing the PDS concentration, reactor temperature, and reaction time resulted in straightforward, but not always sufficient, improvements in both recovery and *δ* ^13^C values. In contrast, modifying the H_3_PO_4_ concentration or adding AgNO_3_ led to less predictable results, likely due to more fundamental shifts in the reaction mechanics, with results that were highly dependent on the specific compound in question. Finally, known methods for evaluating LC‐IRMS oxidation efficiency were combined with our insights from this case study to propose a workflow for method developers faced with the challenge of incomplete oxidation of a novel analyte.

Future instrumental developments could address current challenges. The analysis of stable carbon isotopes has been successfully conducted in a noncommercial conversion interface utilizing photocatalytic laser‐activated combustion, which permits the use of organic eluents [41]. HPLC has also been coupled to a high‐temperature Pt‐catalyzed combustion interface, showing high conversion rates and accurate carbon and nitrogen isotope ratios for caffeine [42]. Furthermore, the direct compound‐ and even position‐specific isotope analysis of polar, thermally labile compounds by Orbitrap mass spectrometers in combination with soft ionization methods is becoming an increasingly important area of research [43].

## Author Contributions

**Felix Niemann:** conceptualization, methodology, formal analysis, investigation, visualization, writing – original draft. **Annika Gruhlke:** conceptualization, methodology, formal analysis, investigation, visualization, writing – review and editing. **Maik A. Jochmann:** conceptualization, methodology, writing – review and editing, supervision. **Torsten C. Schmidt:** conceptualization, methodology, supervision, writing – review and editing.

## Conflicts of Interest

The authors declare no conflicts of interest.

### Peer Review

The peer review history for this article is available at https://www.webofscience.com/api/gateway/wos/peer‐review/10.1002/rcm.10067.

## Supporting information


**Figure S1.** Two‐point calibration obtained by EA‐IRMS measurements of USGS40 and USGS41a.
**Table S1.** Normalized *δ* ^13^C_EA‐IRMS_ of all proxy compounds and standards used to determine LC‐IRMS oxidation efficiency.
**Figure S2.** *δ* ^13^C‐values vs. CO_2_ laboratory working gas of candidate reference materials oxalic acid (black) and NaHCO_3_ (red). The effect of all tested experimental conditions and concentration is presented. Dotted lines indicate the respective mean value.
**Figure S3.** IRMS Peak areas of candidate reference materials oxalic acid (black) and NaHCO_3_ (red). The effect of all tested experimental conditions and concentration is presented.
**Table S2.** Measured pH values of the LC‐IRMS interface eluent for different instrumental conditions. Standard conditions are arbitrarily chosen and refer to 500, 50 and 50 μL min^−1^ flow of eluent, PDS oxidant and acid with concentrations of 100 g L^−1^ PDS and 1.5 M H_3_PO_4_, respectively.
**Table S3.** IRMS backgrounds for all tested instrumental conditions. The resistance of the Faraday cup for *m/z* 32 and *m/z* 44 is 3·10^8^ Ω.
**Figure S4.** Linear regressions of peak areas obtained by IRMS. The red *y*‐axis shows the *δ* ^13^C values relative to the expected EA‐IRMS values.
**Table S4.** Coefficients of determination (*R* ^2^) for linear regressions carried out under different LC‐IRMS oxidation conditions.

## Data Availability

The data that support the findings of this study are available from the corresponding author upon reasonable request.

## References

[1] M. Elsner and G. Imfeld , “Compound‐Specific Isotope Analysis (CSIA) of Micropollutants in the Environment—Current Developments and Future Challenges,” Current Opinion in Biotechnology 41 (2016): 60–72, 10.1016/j.copbio.2016.04.014. 27340797

[2] M. Blessing and N. Baran , “A Review on Environmental Isotope Analysis of Aquatic Micropollutants: Recent Advances, Pitfalls and Perspectives,” TrAC, Trends in Analytical Chemistry 157 (2022): 116730, 10.1016/j.trac.2022.116730.

[3] M. Krummen , A. W. Hilkert , D. Juchelka , A. Duhr , H. J. Schlüter , and R. Pesch , “A New Concept for Isotope Ratio Monitoring Liquid Chromatography/Mass Spectrometry,” Rapid Communications in Mass Spectrometry 18, no. 19 (2004): 2260–2266, 10.1002/rcm.1620. 15384146

[4] L. Elflein and K.‐P. Raezke , “Improved Detection of Honey Adulteration by Measuring Differences Between ^13^C/^12^C Stable Carbon Isotope Ratios of Protein and Sugar Compounds With a Combination of Elemental Analyzer‐Isotope Ratio Mass Spectrometry and Liquid Chromatography‐Isotope Ratio Mass Spectrometry (*δ* ^13^C‐EA/LC‐IRMS),” Apidologie 39, no. 5 (2008): 574–587, 10.1051/apido:2008042.

[5] H. Kawashima , M. Suto , and N. Suto , “Stable Carbon Isotope Ratios for Organic Acids in Commercial Honey Samples,” Food Chemistry 289 (2019): 49–55, 10.1016/j.foodchem.2019.03.053. 30955640

[6] J. P. Godin , J. Hau , L. B. Fay , and G. Hopfgartner , “Isotope Ratio Monitoring of Small Molecules and Macromolecules by Liquid Chromatography Coupled to Isotope Ratio Mass Spectrometry,” Rapid Communications in Mass Spectrometry 19, no. 18 (2005): 2689–2698, 10.1002/rcm.2117. 16124031

[7] S. Willach , H. V. Lutze , K. Eckey , et al., “Direct Photolysis of Sulfamethoxazole Using Various Irradiation Sources and Wavelength Ranges ‐ Insights From Degradation Product Analysis and Compound‐Specific Stable Isotope Analysis,” Environmental Science and Technology 52, no. 3 (2018): 1225–1233, 10.1021/acs.est.7b04744. 29303258

[8] D. M. Kujawinski , L. Zhang , T. C. Schmidt , and M. A. Jochmann , “When Other Separation Techniques Fail: Compound‐Specific Carbon Isotope Ratio Analysis of Sulfonamide Containing Pharmaceuticals by High‐Temperature‐Liquid Chromatography‐Isotope Ratio Mass Spectrometry,” Analytical Chemistry 84, no. 18 (2012): 7656–7663, 10.1021/ac300116w. 22880688

[9] P. R. Martin , D. Buchner , M. A. Jochmann , and S. B. Haderlein , “Stable Carbon Isotope Analysis of Polyphosphonate Complexing Agents by Anion Chromatography Coupled to Isotope Ratio Mass Spectrometry: Method Development and Application,” Analytical and Bioanalytical Chemistry 412 (2020): 4827–4835, 10.1007/s00216-019-02251-w. 31813019

[10] D. A. Abaye , D. J. Morrison , and T. Preston , “Strong Anion Exchange Liquid Chromatographic Separation of Protein Amino Acids for Natural ^13^C‐Abundance Determination by Isotope Ratio Mass Spectrometry,” Rapid Communications in Mass Spectrometry 25, no. 3 (2011): 429–435, 10.1002/rcm.4844. 21213362

[11] M. Perini and L. Bontempo , “Liquid Chromatography Coupled to Isotope Ratio Mass Spectrometry (LC‐IRMS): A Review,” TrAC, Trends in Analytical Chemistry 147 (2022): 116515, 10.1016/j.trac.2021.116515.

[12] D. R. Lide , CRC Handbook of Chemistry and Physics, vol. 85 (CRC press, 2004).

[13] W. Peng , Y. Dong , Y. Fu , et al., “Non‐radical Reactions in Persulfate‐Based Homogeneous Degradation Processes: A Review,” Chemical Engineering Journal 421 (2021): 127818, 10.1016/j.cej.2020.127818.

[14] Y. Ding , X. Wang , L. Fu , et al., “Nonradicals Induced Degradation of Organic Pollutants by Peroxydisulfate (PDS) and Peroxymonosulfate (PMS): Recent Advances and Perspective,” Science of the Total Environment 765 (2021): 142794, 10.1016/j.scitotenv.2020.142794. 33129538

[15] D. Köster , I. M. Sanchez Villalobos , M. A. Jochmann , W. A. Brand , and T. C. Schmidt , “New Concepts for the Determination of Oxidation Efficiencies in Liquid Chromatography–Isotope Ratio Mass Spectrometry,” Analytical Chemistry 91, no. 8 (2019): 5067–5073, 10.1021/acs.analchem.8b05315. 30892863

[16] D. A. Armstrong , R. E. Huie , W. H. Koppenol , et al., “Standard Eelectrode Ppotentials Iinvolving Rradicals in Aaqueous Solution: Iinorganic Rradicals (IUPAC Technical Report),” Pure and Applied Chemistry 87, no. 11–12 (2015): 1139–1150, 10.1515/pac-2014-0502.

[17] C. Liang and H.‐W. Su , “Identification of Sulfate and Hydroxyl Radicals in Thermally Activated Persulfate,” Industrial and Engineering Chemistry Research 48, no. 11 (2009): 5558–5562, 10.1021/ie9002848.

[18] M. Spiro , “The Standard Potential of the Peroxosulphate/Sulphate Couple,” Electrochimica Acta 24, no. 3 (1979): 313–314, 10.1016/0013-4686(79)85051-3.

[19] P. Maruthamuthu and P. Neta , “Phosphate Radicals. Spectra, Acid‐Base Equilibriums, and Reactions With Inorganic Compounds,” Journal of Physical Chemistry 82, no. 6 (1978): 710–713, 10.1021/j100495a019.

[20] N. Li , S. Wu , H. Dai , et al., “Thermal Activation of Persulfates for Organic Wastewater Purification: Heating Modes, Mechanism and Influencing Factors,” Chemical Engineering Journal 450 (2022): 137976, 10.1016/j.cej.2022.137976.

[21] N. M. Beylerian , L. R. Vardanyan , R. S. Harutyunyan , and R. L. Vardanyan , “Kinetics and Mechanism of Potassium Persulfate Decomposition in Aqueous Solutions Studied by a Gasometric Method,” Macromolecular Chemistry and Physics 203, no. 1 (2002): 212–218, 10.1002/1521-3935(20020101)203:1<212::AID-MACP212>3.0.CO;2-3.

[22] F. Minisci , A. Citterio , and C. Giordano , “Electron‐Transfer Processes: Peroxydisulfate, a Useful and Versatile Reagent in Organic Chemistry,” Accounts of Chemical Research 16, no. 1 (1983): 27–32, 10.1021/ar00085a005.

[23] G. Fang , X. Cong , G. Zanoni , Q. Liu , and X. Bi , “Silver‐Based Radical Reactions: Development and Insights,” Advanced Synthesis and Catalysis 359, no. 9 (2017): 1422–1502, 10.1002/adsc.201601179.

[24] T. Gilevska , M. Gehre , and H. H. Richnow , “Performance of the Wet Oxidation Unit of the HPLC Isotope Ratio Mass Spectrometry System for Halogenated Compounds,” Analytical Chemistry 86, no. 15 (2014): 7252–7257, 10.1021/ac501174d. 24975492

[25] L. Zhang , D. M. Kujawinski , M. A. Jochmann , and T. C. Schmidt , “High‐Temperature Reversed‐Phase Liquid Chromatography Coupled to Isotope Ratio Mass Spectrometry,” Rapid Communications in Mass Spectrometry 25, no. 20 (2011): 2971–2980, 10.1002/rcm.5069. 21953951

[26] L. Zhang , D. M. Kujawinski , E. Federherr , T. C. Schmidt , and M. A. Jochmann , “Caffeine in Your Drink: Natural or Synthetic?,” Analytical Chemistry 84, no. 6 (2012): 2805–2810, 10.1021/ac203197d. 22339647

[27] S. Reinnicke , A. Bernstein , and M. Elsner , “Small and Reproducible Isotope Effects During Methylation With Trimethylsulfonium Hydroxide (TMSH): A Convenient Derivatization Method for Isotope Analysis of Negatively Charged Molecules,” Analytical Chemistry 82, no. 5 (2010): 2013–2019, 10.1021/ac902750s. 20143836

[28] E. Federherr , C. Cerli , F. Kirkels , et al., “A Novel High‐Temperature Combustion Based System for Stable Isotope Analysis of Dissolved Organic Carbon in Aqueous Samples. I: Development and Validation,” Rapid Communications in Mass Spectrometry 28, no. 23 (2014): 2559–2573, 10.1002/rcm.7052. 25366403

[29] S. Cueto Diaz , J. R. Encinar , A. Sanz‐Medel , and J. I. G. Alonso , “Liquid Chromatography, Chemical Oxidation, and Online Carbon Isotope Dilution Mass Spectrometry as a Universal Quantification System for Nonvolatile Organic Compounds,” Analytical Chemistry 85, no. 3 (2013): 1873–1879, 10.1021/ac3032542. 23252800

[30] F. Niemann , A. Gruhlke , K. Kerpen , M. A. Jochmann , and T. C. Schmidt , “Insight Into Imidacloprid Degradation Through Compound Specific Carbon Isotope Analysis and High‐Resolution Mass Spectrometry,” ACS ES&T Water 4, no. 12 (2024): 5180–5993, 10.1021/acsestwater.4c00552.

[31] H. Qi , T. B. Coplen , H. Geilmann , W. A. Brand , and J. K. Böhlke , “Two New Organic Reference Materials for *δ* ^13^C and *δ* ^15^N Measurements and a New Value for the *δ* ^13^C of NBS 22 Oil,” Rapid Communications in Mass Spectrometry 17, no. 22 (2003): 2483–2487, 10.1002/rcm.1219. 14608617

[32] H. Qi , T. B. Coplen , S. J. Mroczkowski , et al., “A New Organic Reference Material, l‐Glutamic Acid, USGS41a, for *δ* ^13^C and *δ* ^15^N Measurements − A Replacement for USGS41,” Rapid Communications in Mass Spectrometry 30, no. 7 (2016): 859–866, 10.1002/rcm.7510. 26969927

[33] Scientific TF. Finnigan LC IsoLink LC‐IRMS Interface Operating Manual Revision C‐1156140. In:2004.

[34] E. Hettmann , W. A. Brand , and G. Gleixner , “Improved Isotope Ratio Measurement Performance in Liquid Chromatography/Isotope Ratio Mass Spectrometry by Removing Excess Oxygen,” Rapid Communications in Mass Spectrometry 21, no. 24 (2007): 4135–4141, 10.1002/rcm.3304. 18041012

[35] J. P. Godin High‐Precision ^13^C Isotopic Analyses in Life Sciences by Gas and Liquid Chromatography Coupled to Isotope Ratio Mass Spectrometry: Life Sciences Mass Spectrometry, University of Geneva; (2008).

[36] D. Köster , M. Jochmann , H. Lutze , and T. Schmidt , “Monitoring of the Total Carbon and Nitrogen Balance During the Mineralization of Nitrogen Containing Compounds by Heat Activated Persulfate,” Chemical Engineering Journal 367 (2019): 160–168, 10.1016/j.cej.2019.02.115.

[37] O. Van Cleemput and A. H. Samater , “Nitrite in Soils: Accumulation and Role in the Formation of Gaseous N Compounds,” Fertilizer Research 45 (1995): 81–89, 10.1007/BF00749884.

[38] P. D. Goulden and D. H. Anthony , “Kinetics of Uncatalyzed Peroxydisulfate Oxidation of Organic Material in Fresh Water,” Analytical Chemistry 50, no. 7 (1978): 953–958, 10.1021/ac50029a032.

[39] M. L. Dell'Arciprete , C. J. Cobos , J. P. Furlong , D. O. Mártire , and M. C. Gonzalez , “Reactions of Sulphate Radicals With Substituted Pyridines: A Structure–Reactivity Correlation Analysis,” ChemPhysChem 8, no. 17 (2007): 2498–2505, 10.1002/cphc.200700456. 17957815

[40] X. Duan , X. Niu , J. Gao , S. Wacławek , L. Tang , and D. D. Dionysiou , “Comparison of Sulfate Radical With Other Reactive Species,” Current Opinion in Chemical Engineering 38 (2022): 100867, 10.1016/j.coche.2022.100867.

[41] H. J. Tobias , A. Jones , T. R. Saunders , and J. T. Brenna , “Liquid Chromatography Coupled to Isotope Ratio Mass Spectrometry Expanded to Include Organic Mobile Phases,” Analytical Chemistry 96, no. 19 (2024): 7348–7352, 10.1021/acs.analchem.3c03583. 38696329

[42] E. Federherr , S. Willach , N. Roos , L. Lange , K. Molt , and T. Schmidt , “A Novel High‐Temperature Combustion Interface for Compound‐Specific Stable Isotope Analysis of Carbon and Nitrogen via High‐Performance Liquid Chromatography/Isotope Ratio Mass Spectrometry,” Rapid Communications in Mass Spectrometry 30, no. 7 (2016): 944–952, 10.1002/rcm.7524. 26969937

[43] K. Kantnerová , N. Kuhlbusch , D. Juchelka , A. Hilkert , S. Kopf , and C. Neubauer , “A Guide to Precise Measurements of Isotope Abundance by ESI‐Orbitrap MS,” Nature Protocols 457 (2024): 1–32, 10.1016/j.ijms.2020.116410. 38654136

