# Supplementary material for: LC‐IRMS Persulfate Oxidation: Case Study on Neonicotinoid‐Related Structures
Source: Rapid Commun Mass Spectrom. 2025 May 12;39(16):e10067. doi: 10.1002/rcm.10067 (PMC12068030; doi:10.1002/rcm.10067)
Supplement: Supplementary file 1 — Figure S1. Two‐point calibration obtained by EA‐IRMS measurements of USGS40 and USGS41a. Table S1. Normalized δ 13CEA‐IRMS of all proxy compounds and standards used to determine LC‐IRMS oxidation efficiency. Figure S2. δ 13C‐values vs. CO2 laboratory working gas of candidate reference materials oxalic acid (black) and NaHCO3 (red). The effect of all tested experimental conditions and concentration is presented. Dotted lines indicate the respective mean value. Figure S3. IRMS Peak areas of candidate reference materials oxalic acid (black) and NaHCO3 (red). The effect of all tested experimental conditions and concentration is presented. Table S2. Measured pH values of the LC‐IRMS interface eluent for different instrumental conditions. Standard conditions are arbitrarily chosen and refer to 500, 50 and 50 μL min−1 flow of eluent, PDS oxidant and acid with concentrations of 100 g L−1 PDS and 1.5 M H3PO4, respectively. Table S3. IRMS backgrounds for all tested instrumental conditions. The resistance of the Faraday cup for m/z 32 and m/z 44 is 3·108 Ω. Figure S4. Linear regressions of peak areas obtained by IRMS. The red y‐axis shows the δ 13C values relative to the expected EA‐IRMS values. Table S4. Coefficients of determination (R 2) for linear regressions carried out under different LC‐IRMS oxidation conditions. [file RCM-39-e10067-s001.docx]

LC-IRMS Persulfate Oxidation: Case Study on Neonicotinoid-Related Structures

Felix Niemann^1^, Annika Gruhlke^1^, Maik A. Jochmann^1,2*^, Torsten C. Schmidt^1,2,3^

^1^University of Duisburg-Essen, Faculty of Chemistry, Instrumental Analytical Chemistry, Universitätsstraße 5, D-45141 Essen, Germany

^2^Centre for Water and Environmental Research (ZWU), Universitätsstraße 5, D-45141 Essen, Germany

^3^IWW Water Centre, Moritzstraße 26, D-45476 Mülheim an der Ruhr, Germany

**Keywords:** LC-IRMS, Oxidation Interface, Persulfate, Neonicotinoids

**Section S1. Chemicals and Reagents**

**Chemicals.** Carbon dioxide (99.9995%), helium (99.9990%), and synthetic air (20.5 ± 0.5% O_2_) were obtained from Air Liquide (Düsseldorf, Germany). Urea (≥ 99.0%) was obtained from AppliChem (Darmstadt, Germany). Imidacloprid-urea (97%) was obtained from BLD Pharmatech (Reinbek, Germany). Oxalic acid dihydrate (≥ 99.5%) was obtained from Carl Roth (Karlsruhe, Germany). Sodium nitrate (≥ 99.5%) and sodium persulfate (≥ 99.0%) were obtained from Honeywell (Offenbach, Germany). Acetanilide was obtained from IVA Analysentechnik (Meerbusch, Germany). Sodium bicarbonate (≥ 99.9%) was obtained from KMF Laborchemie (Lohmar, Germany). *Ortho*-phosphoric acid (85%) was obtained from Merck (Darmstadt, Germany). Isotope reference materials USGS40 and 41a were obtained from Reston Stable Isotope Laboratory (Reston, USA). Clothianidin (96%), imidacloprid (≥ 95.0%), imidaclothiz (98 %) and N-nitroimidazolidin-2-imine (≥ 98%) were obtained from Santa Cruz Biotechnology (Heidelberg, Germany). 2-imidazolidone, 6-chloropyridine-3-carboxylic acid (99%), ethylendiamine (≥ 99%), guanidine hydrochloride (≥ 99%), nitroguanidine (80% desensitized with H_2_O) and silver nitrate (≥ 99.0%) were obtained from Sigma-Aldrich (Taufkirchen, Germany).

**Preparation and Storage of Standards.** To obtain accurate carbon recoveries by LC-IRMS, it is essential that the standards are weighed with precision. It was necessary to ensure that the reference substance, NaHCO₃, was free of water to calculate correct recoveries of all model substances. Moreover, GUA is hygroscopic, and NGUA is desensitized with water; therefore, they were subjected to a drying process using a Vacutherm VT 6025 vacuum drier (Thermo Fisher Scientific, Bremen, Germany) and stored in a desiccator. The drying process was conducted at 60 °C and 50 mbar for a period of four hours. Thereafter, the samples were stored in an evacuated desiccator. It was essential to store the NaHCO_3_ solutions in a gas-tight container to prevent the dissolution of atmospheric CO_2_. All solutions were stored gas-tight in the dark at approximately 7 °C.

**Section S2. EA-IRMS Measurements of Proxy Compounds and Standards**

To determine oxidation-independent *δ*^13^C values for all proxy compounds and standards, elemental analyzer isotope ratio mass spectrometry (EA-IRMS) measurements were performed. They were weighed into tin cups and then folded tightly to remove air. A laboratory acetanilide standard was used to monitor instrument stability. The international reference materials USGS40 (*δ*^13^C_VPDB_ = -26.39 ± 0.04‰)^1^, and USGS41a (*δ*^13^C_VPDB_ = 36.55 ± 0.08‰)^2^ were measured along with all other samples to determine the normalized *δ*^13^C_EA-IRMS_ values of all compounds using a two-point calibration (Figure S1). As a blank control, empty tin cups were folded and measured. The *δ*^13^C_EA-IRMS_ values of all proxy compounds resulting from the linear calibration are presented in Table S1.


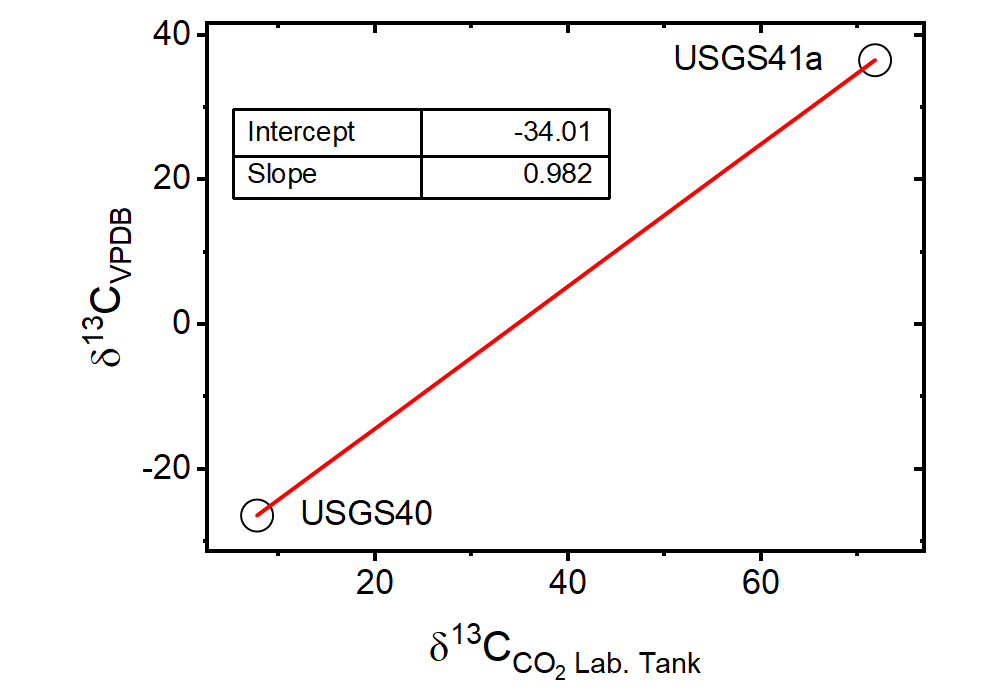


**Figure S1.** 2-point calibration obtained by EA-IRMS measurements of USGS40 and USGS41a.

**Table S1.** Normalized *δ*^13^C_EA-IRMS_ of all proxy compounds and standards used to determine LC-IRMS oxidation efficiency.

| **Compound** | *δ***^13^C_EA-IRMS_ [‰]** |
| --- | --- |
| 2-Imidazolidone | -32.04 ± 0.03 |
| 6-Chloropyridine-3-carboxylic acid | -34.22 ± 0.07 |
| Clothianidin | -27.9 ± 0.2 |
| Desnitro-imidacloprid hydrochloride | -32.83 ± 0.05 |
| Ethylenediamine | -22.26 ± 0.56 |
| Guanidine hydrochloride | -24.9 ± 0.3 |
| Imidacloprid | -31.19 ± 0.05 |
| Imidacloprid urea | -32.6 ± 0.1 |
| Imidaclothiz | -25.2 ± 0.2 |
| N-Nitroimidazolidin-2-imine | -26.7 ± 0.1 |
| Nitroguanidine | -25.3 ± 1.1 |
| Oxalic acid dihydrate | -33.8 ± 1.7 |
| Sodium hydrogen carbonate | -7.44 ± 0.09 |
| Urea | -46.94 ± 0.31 |

**Section S3. Reference Compound Selection for Normalization of LC-IRMS** *δ***^13^C values**

In order to evaluate the performance of the wet combustion interface of the LC-IRMS, it was necessary to identify a suitable, easily oxidizable, or oxidation-independent reference substances that could serve as anchor points for complete carbon recoveries and the VPDB scale. In a preliminary experiment, oxalic acid, Na_2_CO_3_, and NaHCO_3_ were tested as potential candidates for this purpose. Oxalic acid solutions were prepared using oxalic acid dihydrate, which exhibits reduced hygroscopic properties in comparison to its water-free form. Previous studies have also employed small carbonic acids or inorganic carbon for this purpose.^3-5^ However, Na₂CO₃ was excluded due to the dissolution of atmospheric CO₂ into the alkaline solution during storage periods exceeding 1-2 weeks, which resulted in increased peak areas and *δ*^13^C bias. This phenomenon did not occur for less alkaline NaHCO_3_ stored in a gas-tight vessel. Figure S2 illustrates the influence of oxidation conditions on the *δ*^13^C values of the remaining two candidate reference materials, oxalic acid and NaHCO_3_. The isotope ratios of these materials are independent of oxidation conditions, as indicated by a maximum deviation of ± 1‰. This makes them suitable reference materials. Figure S2 illustrates the impact of oxidation conditions on the IRMS peak area of candidate reference compounds. Minor discrepancies in peak areas observed between oxidation conditions for identical quantities of injected carbon, particularly when the total flow rate is modified, can be attributed to the specific condition and are not indicative of inadequate transformation to CO_2_.


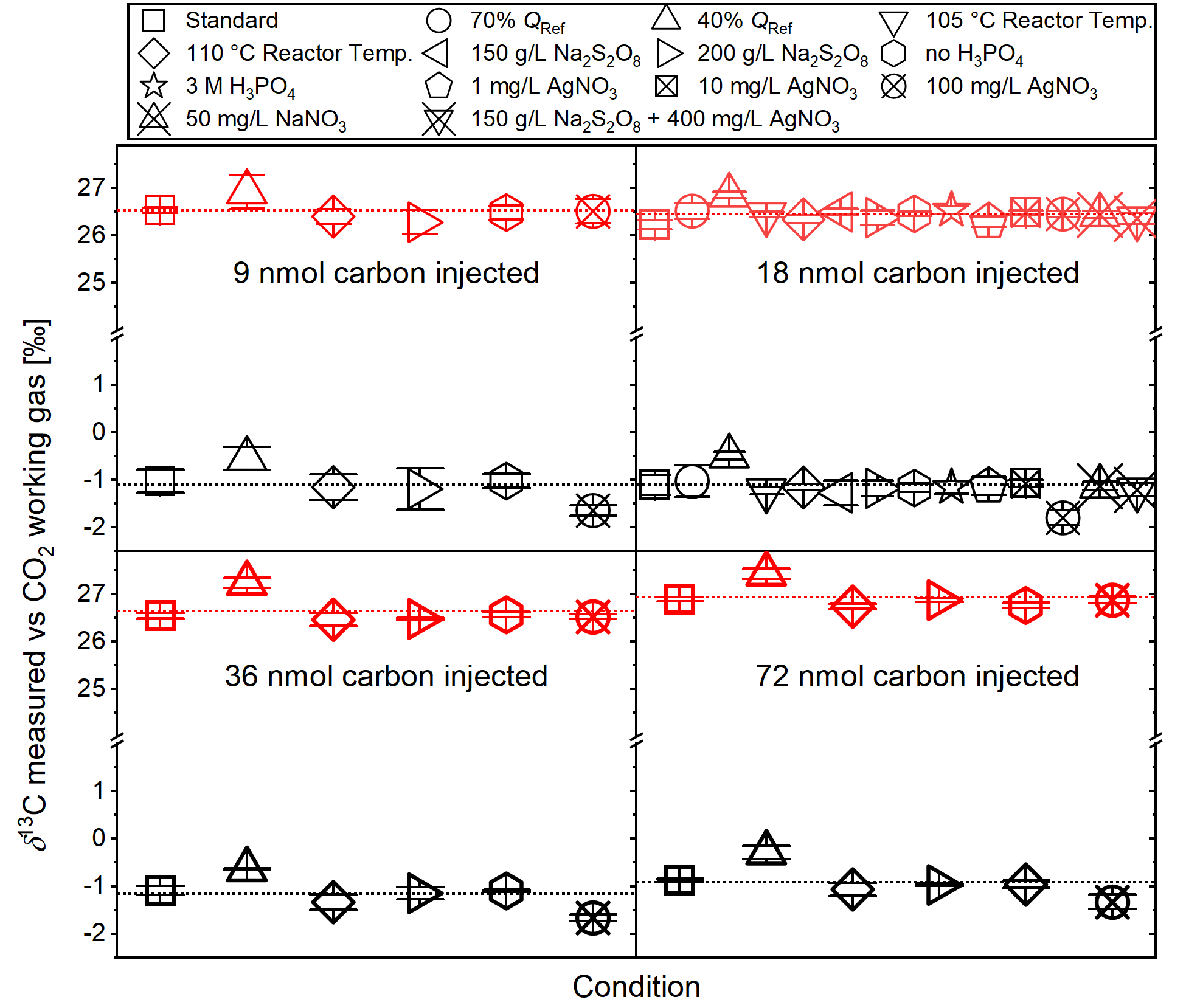


**Figure S2.** *δ*^13^C-values vs. CO_2_ laboratory working gas of candidate reference materials oxalic acid (black) and NaHCO_3_ (red). The effect of all tested experimental conditions and concentration is presented. Dotted lines indicate the respective mean value.


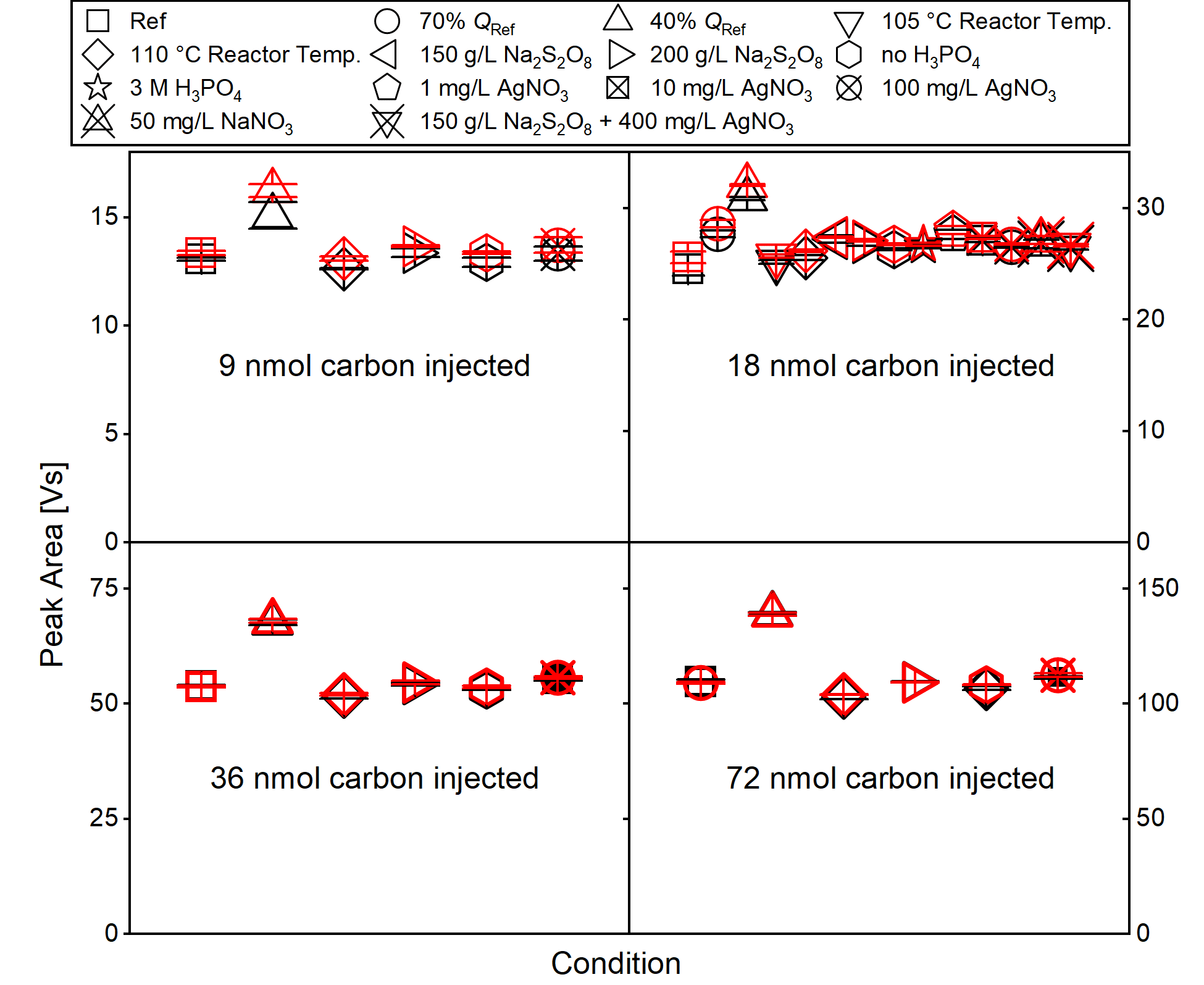


**Figure S3.** IRMS Peak areas of candidate reference materials oxalic acid (black) and NaHCO_3_ (red). The effect of all tested experimental conditions and concentration is presented.

**Section S3. PH-value of Effluent**

PDS oxidation is strongly dependent on pH but pH measurements in the heated reaction zone are not feasible. The pH of the effluent was measured at room temperature and all values are reported in Table S2.

**Table S2.** Measured pH values of the LC-IRMS interface eluent for different instrumental conditions. Standard conditions are arbitrarily chosen and refer to 500, 50 and 50 µL min^-1^ flow of eluent, PDS oxidant and acid with concentrations of 100 g L^-1^ PDS and 1.5 M H_3_PO_4_, respectively.

| Instrumental Conditions | Interface Effluent pH |
| --- | --- |
| Standard | 1.51 |
| 70% *Q*_Ref_ | 1.44 |
| 40% *Q*_Ref_ | 1.46 |
| 105 °C Reactor Temperature | 1.47 |
| 110 °C Reactor Temperature | 1.49 |
| 150 g L^-1^ Na_2_S_2_O_8_ | 1.44 |
| 200 g L^-1^ Na_2_S_2_O_8_ | 1.38 |
| No H_3_PO_4_ | 2.37 |
| 3 M H_3_PO_4_ | 1.25 |
| 1 mg L^-1^ AgNO_3_ | 1.46 |
| 10 mg L^-1^ AgNO_3_ | 1.50 |
| 100 mg L^-1^ AgNO_3_ | 1.46 |

**Section S4. LC-IRMS Background Signals**

Instrumental flow and oxidation conditions affect LC-IRMS backgrounds. High O_2_ backgrounds reduce the lifetime of the IRMS filament, and unstable or high CO_2_ backgrounds can reduce the accuracy of stable carbon isotope measurements or increase the amount of injected carbon required for accurate isotope analysis. Backgrounds as a function of instrumental settings are given in Table S3.

**Table S3.** IRMS backgrounds for all tested instrumental conditions. The resistance of the Faraday cup for *m/z* 32 and *m/z* 44 is 3∙10^8^ Ω.

| c(Na_2_S_2_O_8_) [g L^-1^] | c(H_3_PO_4_) [M] | Catalyst [mg L^-1^] | Flow Eluent [μL min^-1^] | Flow Oxidant/ Acid [μL min^-1^] | Reactor Temp. [°C] | O_2_  [m/z 32]  [V] | CO_2_  [m/z 44] [mV] |
| --- | --- | --- | --- | --- | --- | --- | --- |
| 100 | 1.5 | - | 500 | 50/50 | 100 | 13.3 | 203 |
| 100 | 1.5 | - | 350 | 35/35 | 100 | 11.6 | 115 |
| 100 | 1.5 | - | 200 | 20/20 | 100 | 9.4 | 71 |
| 100 | 1.5 | - | 500 | 50/50 | 105 | 21.6 | 156 |
| 100 | 1.5 | - | 500 | 50/50 | 110 | 36.2 | 157 |
| 150 | 1.5 | - | 500 | 50/50 | 100 | 18.7 | 279 |
| 200 | 1.5 | - | 500 | 50/50 | 100 | 23.7 | 309 |
| 100 | - | - | 500 | 50/50 (H_2_O) | 100 | 9.9 | 134 |
| 100 | 3 | - | 500 | 50/50 | 100 | 14.8 | 151 |
| 100 | 1.5 | 1  (AgNO_3_) | 500 | 50/50 | 100 | 14.7 | 210 |
| 100 | 1.5 | 10  (AgNO_3_) | 500 | 50/50 | 100 | 14.2 | 179 |
| 100 | 1.5 | 100  (AgNO_3_) | 500 | 50/50 | 100 | 13.8 | 187 |

**Section S5. Linearity**

All linear regressions obtained by injecting different carbon amounts of each of the 14 tested probe compounds are presented in Figure S4. The respective coefficients of determination are given in Table S4.

| 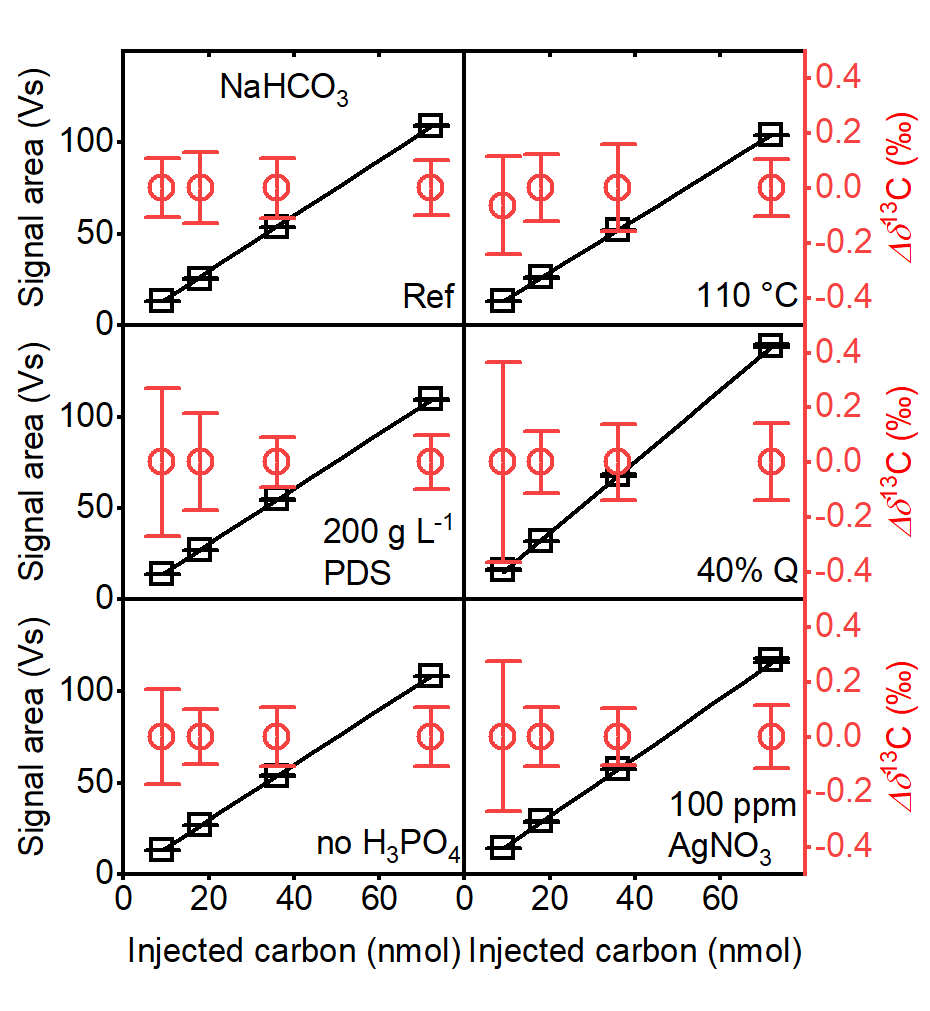 | 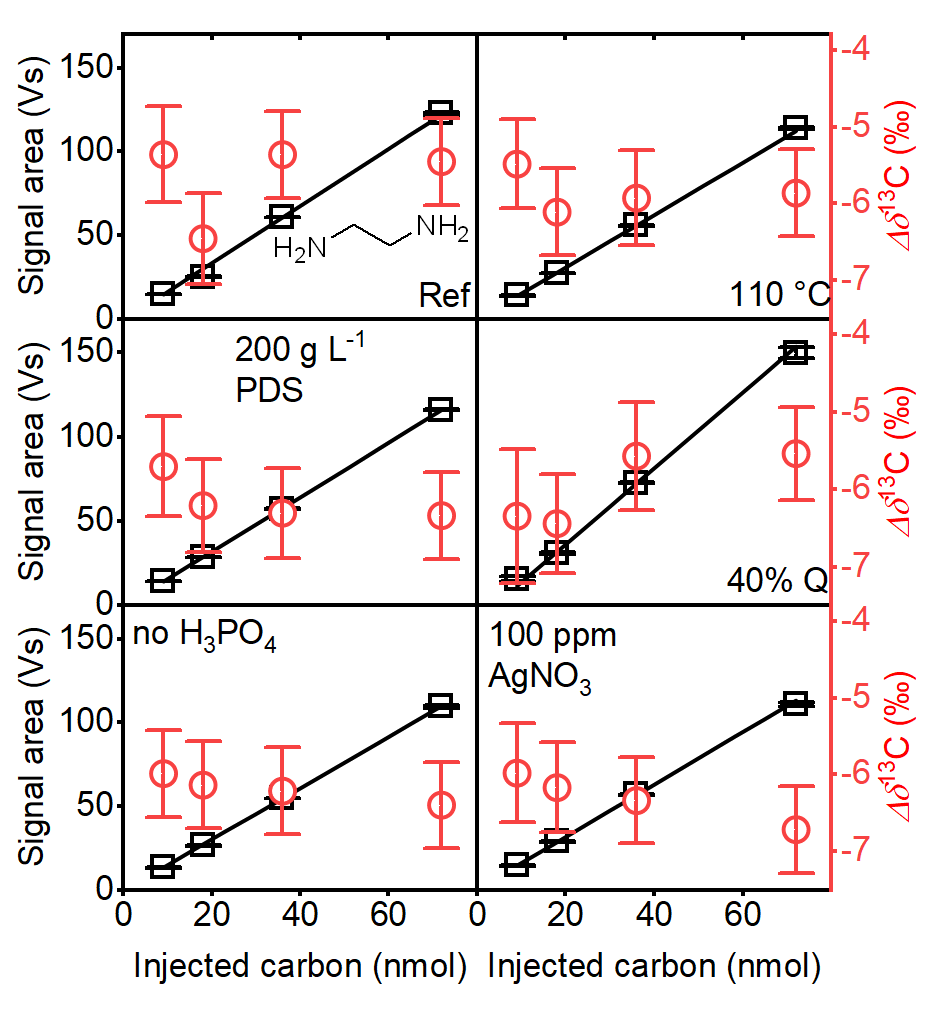 |
| --- | --- |
| 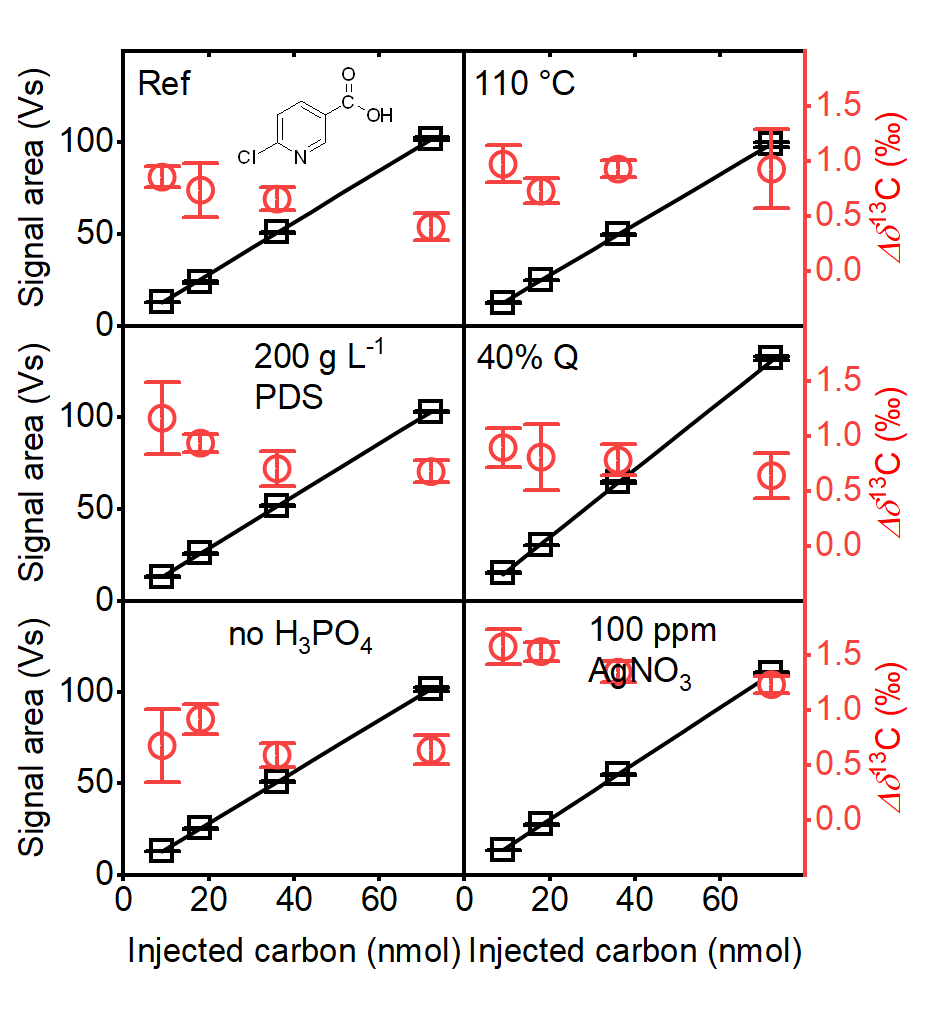 | 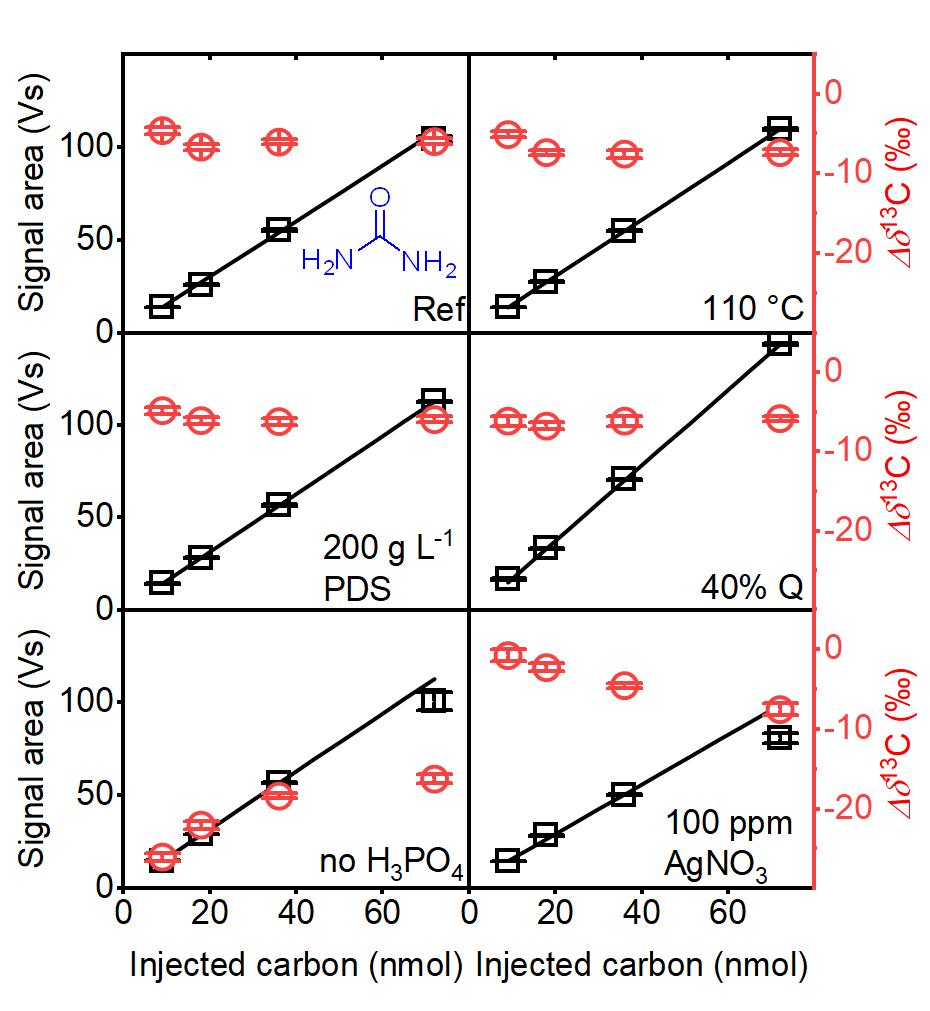 |
| 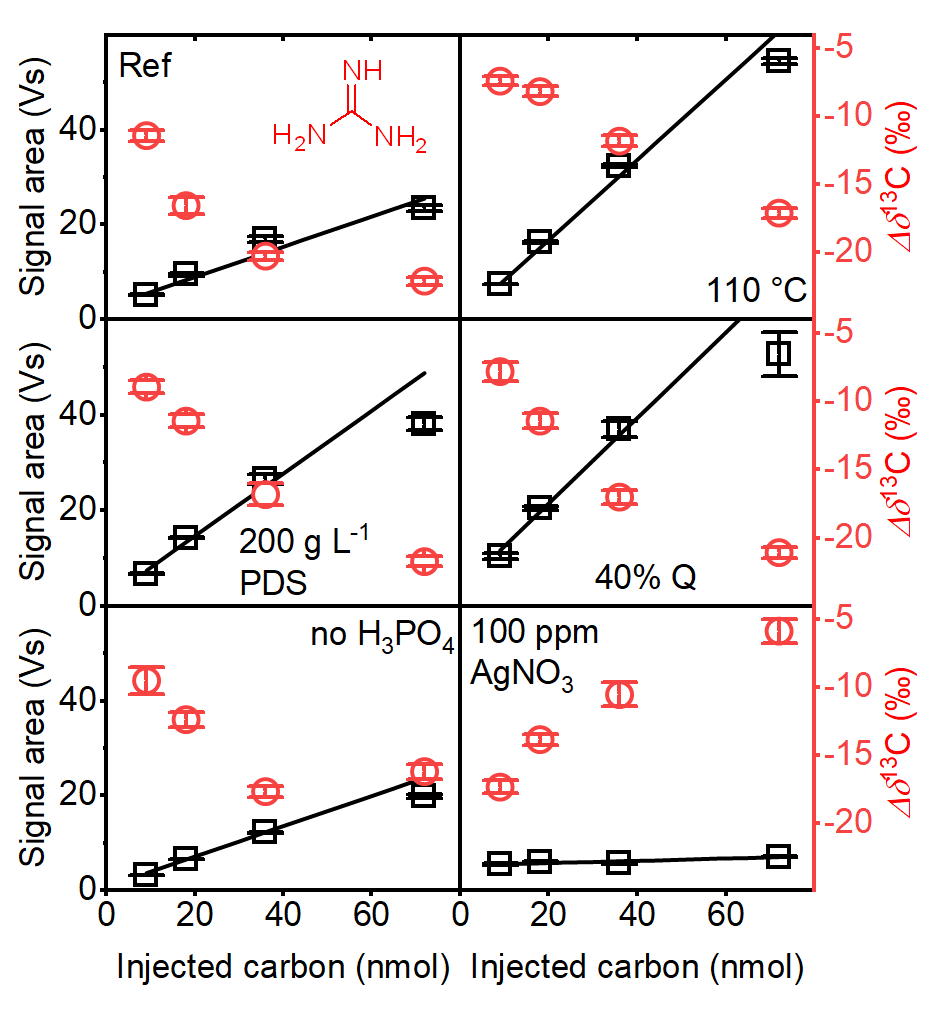 | 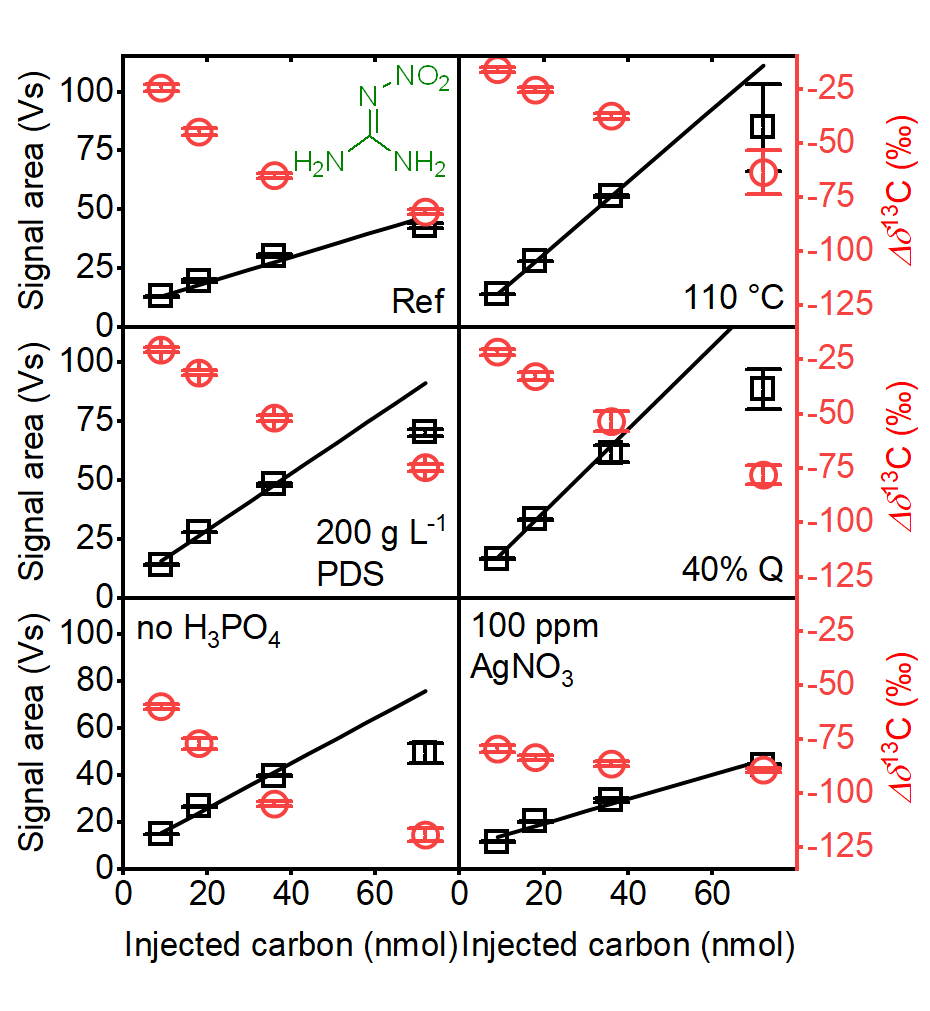 |
| 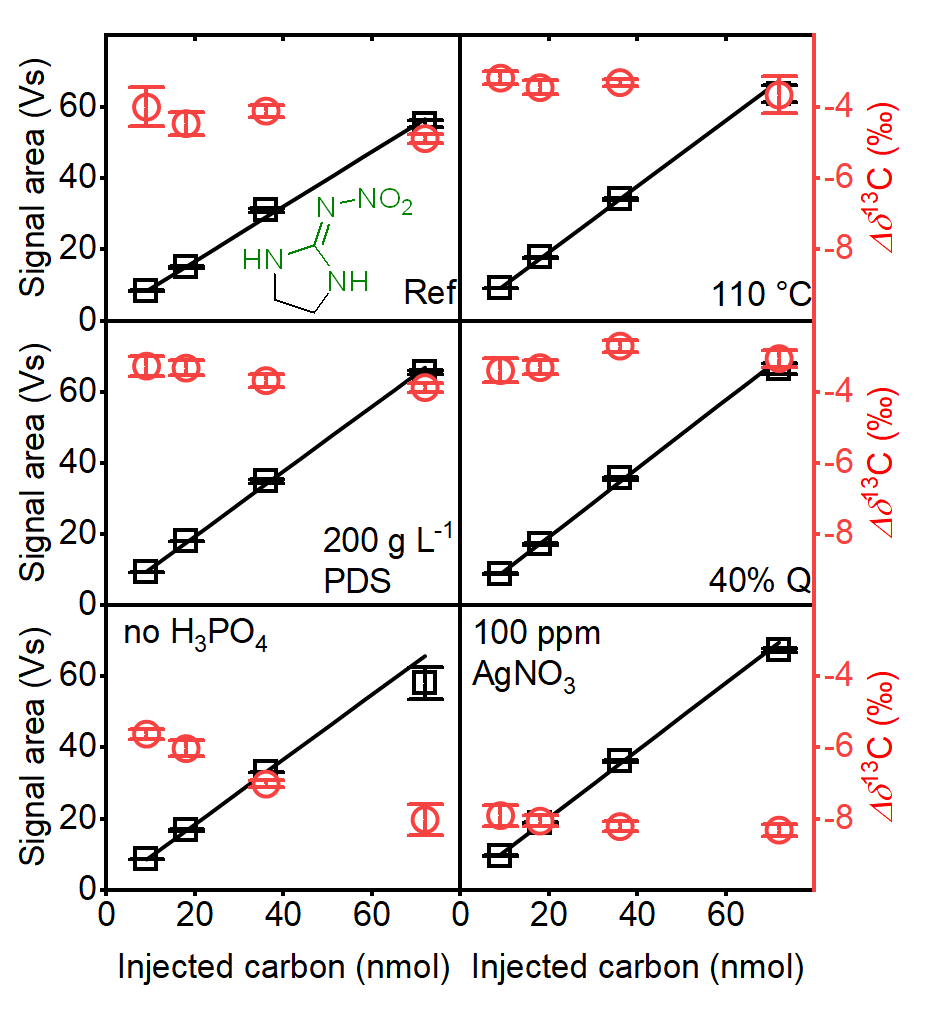 | 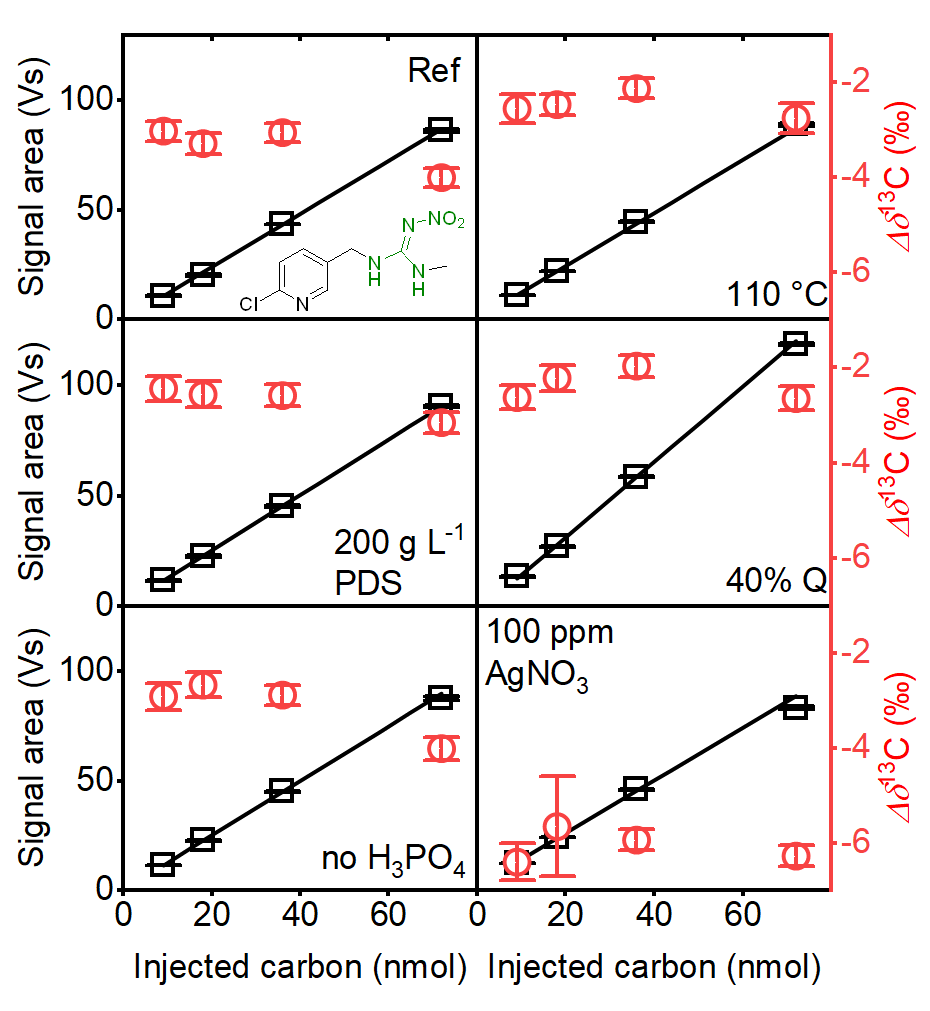 |
| 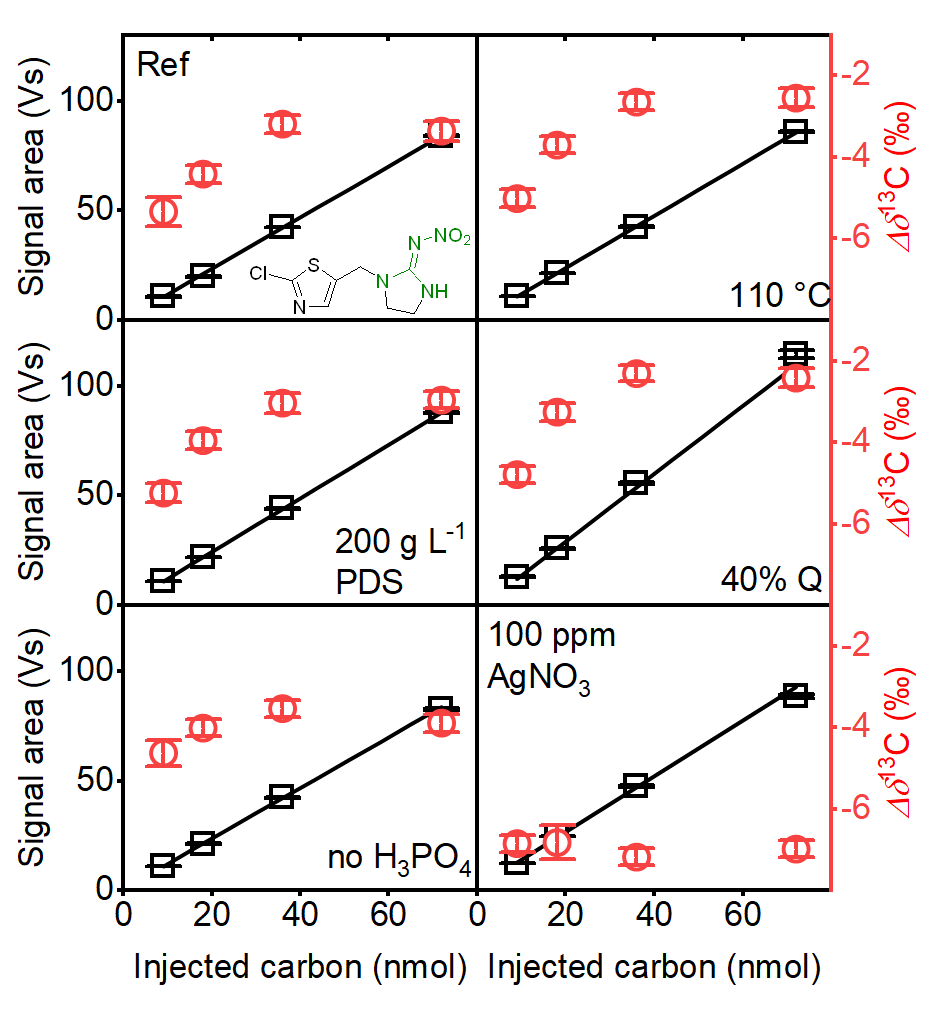 | 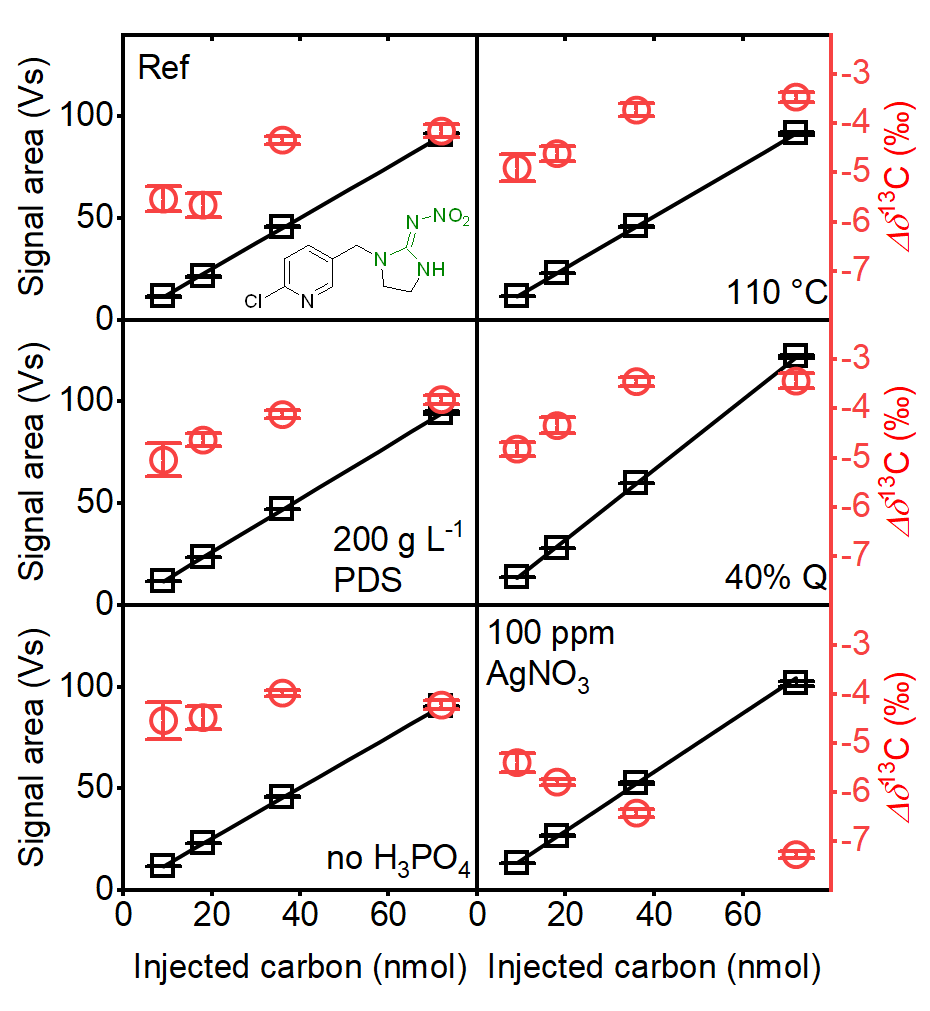 |
| 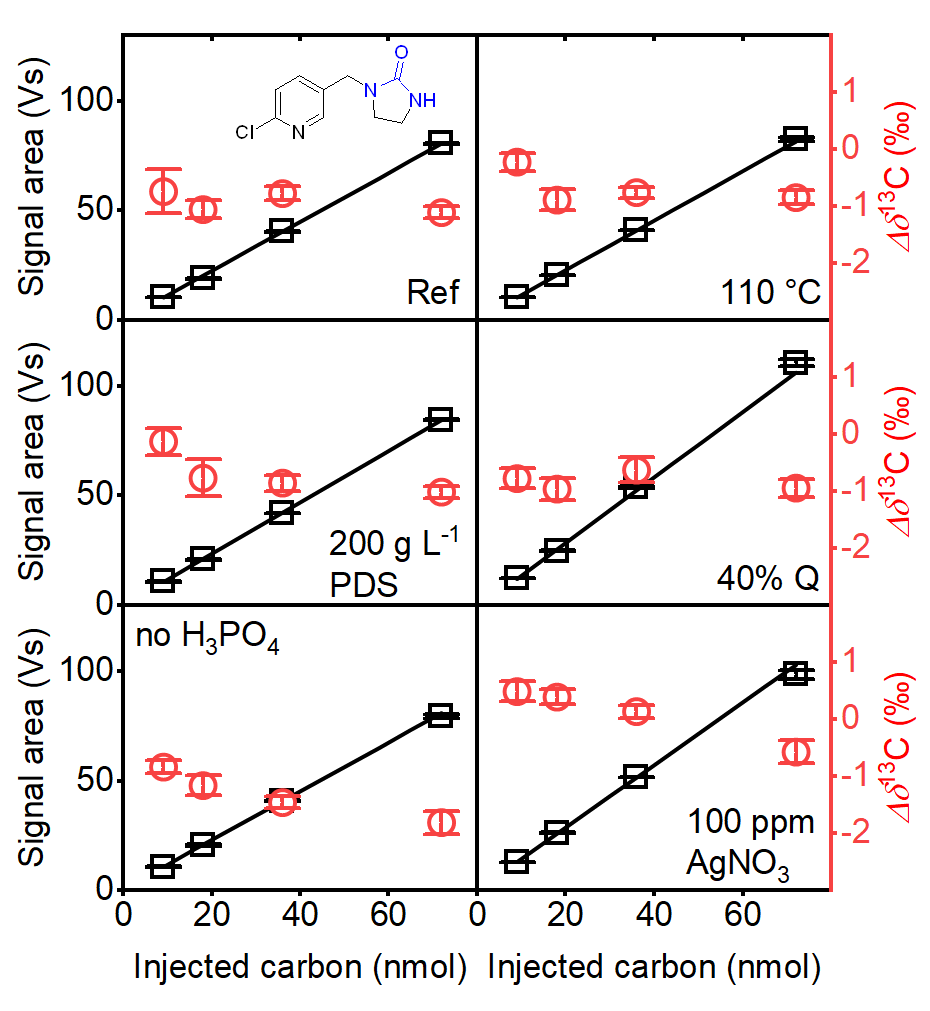 | 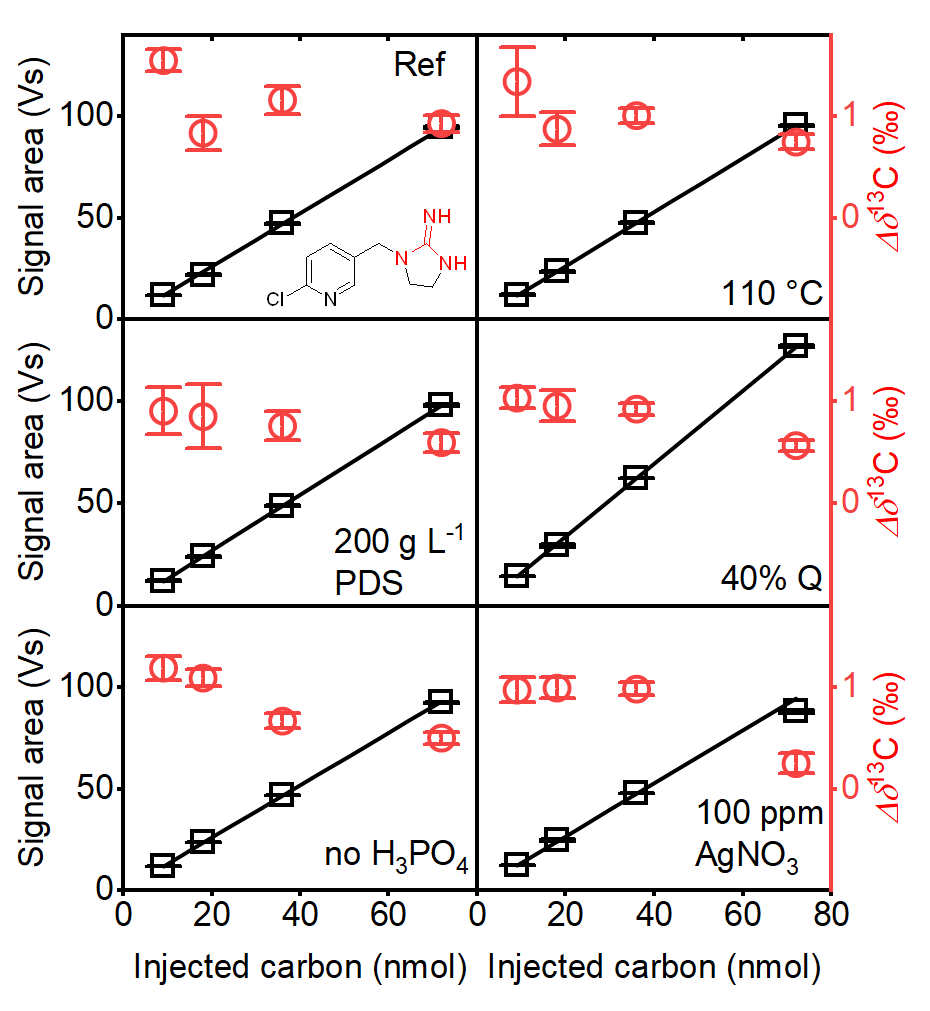 |

**Figure S4.** Linear regressions of peak areas obtained by IRMS. The red y-axis shows the *δ*^13^C values relative to the expected EA-IRMS values.

**Table S4.** Coefficients of determination (R^2^) for linear regressions carried out under different LC-IRMS oxidation conditions.

| **R^2^** |  | | | | | |
| --- | --- | --- | --- | --- | --- | --- |
| **Compound** | **Ref** | **40% *Q*_Ref_** | **110 °C** | **200 g L^-1^ PDS** | **No H_3_PO_4_** | **100 ppm AgNO_3_** |
| NaHCO_3_ | 0.99982 | 0.99845 | 1.00000 | 0.99999 | 0.99999 | 0.99997 |
| IMZO | 0.99908 | 0.99664 | 0.99981 | 0.99180 | 0.96344 | 0.99786 |
| 6-CNA | 0.99943 | 0.99788 | 0.99999 | 1.00000 | 1.00000 | 0.99999 |
| CLO | 0.99976 | 0.99903 | 0.99988 | 0.99999 | 0.99992 | 0.99488 |
| DN-IMI | 0.99908 | 0.99999 | 0.99993 | 0.99994 | 0.99985 | 0.99844 |
| EDA | 0.99831 | 0.99687 | 0.99927 | 0.99996 | 0.99995 | 0.99981 |
| GUA | 0.93392 | 0.92745 | 0.97856 | 0.90450 | 0.98413 | 0.89530 |
| IMI | 0.99824 | 0.99930 | 0.99992 | 0.99997 | 0.99995 | 0.99946 |
| IMI-U | 0.99982 | 0.99492 | 0.99985 | 0.99991 | 0.99996 | 0.99988 |
| IMZ | 0.99943 | 0.99296 | 0.99994 | 1.00000 | 0.99984 | 0.99593 |
| NIZO | 0.99476 | 0.99702 | 0.99914 | 0.9982 | 0.99993 | 0.99639 |
| NGUA | 0.96004 | 0.97536 | 0.99976 | 0.90446 | 0.95747 | 0.91828 |
| Urea | 0.99707 | 0.99943 | 0.99999 | 0.99999 | 0.9996 | 0.98698 |

**References**

(1) Qi, H.; Coplen, T. B.; Geilmann, H.; Brand, W. A.; Böhlke, J. K. Two new organic reference materials for *δ*^13^C and *δ*^15^N measurements and a new value for the *δ*^13^C of NBS 22 oil. *Rapid Communications in Mass Spectrometry* **2003**, *17* (22), 2483-2487. DOI: <https://doi.org/10.1002/rcm.1219> From NLM.

(2) Qi, H.; Coplen, T. B.; Mroczkowski, S. J.; Brand, W. A.; Brandes, L.; Geilmann, H.; Schimmelmann, A. A new organic reference material, l-glutamic acid, USGS41a, for *δ*^13^C and *δ*^15^N measurements − a replacement for USGS41. *Rapid Communications in Mass Spectrometry* **2016**, *30* (7), 859-866. DOI: <https://doi.org/10.1002/rcm.7510>.

(3) Köster, D.; Sanchez Villalobos, I. M.; Jochmann, M. A.; Brand, W. A.; Schmidt, T. C. New Concepts for the Determination of Oxidation Efficiencies in Liquid Chromatography–Isotope Ratio Mass Spectrometry. *Analytical Chemistry* **2019**, *91* (8), 5067-5073. DOI: <https://doi.org/10.1021/acs.analchem.8b05315>.

(4) Gilevska, T.; Gehre, M.; Richnow, H. H. Performance of the Wet Oxidation Unit of the HPLC Isotope Ratio Mass Spectrometry System for Halogenated Compounds. *Analytical Chemistry* **2014**, *86* (15), 7252-7257. DOI: <https://doi.org/10.1021/ac501174d>.

(5) Martin, P. R.; Buchner, D.; Jochmann, M. A.; Haderlein, S. B. Stable carbon isotope analysis of polyphosphonate complexing agents by anion chromatography coupled to isotope ratio mass spectrometry: method development and application. *Analytical and Bioanalytical Chemistry* **2020**, *412*, 4827-4835. DOI: <https://doi.org/10.1007/s00216-019-02251-w>.
